# Does HLA explain the high incidence of childhood-onset type 1 diabetes in the Canary Islands? The role of Asp57 *DQB1* molecules

**DOI:** 10.1186/s12887-024-04983-w

**Published:** 2024-09-06

**Authors:** Yeray Nóvoa-Medina, Itahisa Marcelino-Rodriguez, Nicolás M. Suárez, Marta Barreiro-Bautista, Eva Rivas-García, Santiago Sánchez-Alonso, Gema González-Martínez, Sofía Quinteiro-González, Ángela Domínguez, María Cabrera, Sara López, Svetlana Pavlovic, Carlos Flores, Gran Canarian Diabetes and Obesity Research Group, Carlota Rodriguez-Benitez, Héctor Ageno-Alemán, Cristina Perera-Hernández, Catalina de Elejabeitia-Cortezo, Nieves Franco-Mateu, Ana María Rodríguez Gonzalez, Victor Manuel Leon-Olmo, Fátima Aitoutouhen-Infante, Sade Pérez-López, Saula del Pino Alonso-Falcón, Acoraida Bolaños-Alzola, Zeltia García-Suárez, Inés Perdomo-Delgado, Sara Ayala-Martínez, Laura Valenzuela-Alvarez, Elena Caballero-Estupiñán, Celia Rúa-Figueroa, Claudia Travieso-Hermoso, Yaiza García-Delgado, Pablo Azcoitia, Sara Quintana Arroyo, Carlos Rodríguez, Yaiza López-Plasencia, Nuria Pérez-Martín, Rosa María Sánchez-Hernández, María José López-Madrazo, Alejandro Déniz, Rossella Tozzi, Mauro Boronat-Cortés, Carmen Valverde-Tercedor, Garlene Zamora-Zamorano, Roberto Jiménez-Monzón, Luisa Hernández-Baeza, Verónica Dávila-Batista, Yaiza Gil, Oliver Gil Jorge, Romina Soage-Villegas, Sofia Bueno-Montoro, Aitana Guanche-Sicilia, Brenda Santos-Morán, Jesús Santana-Medina, Sofía Ojeda-Elías, Beatriz Melian-Cordovez, Marina Corona-Lopez, Marta Macías-Dolz, Saray Betancort-Avero, Samuel Rodriguez-Déniz, Ana Puga-Morales, Rose Bowler-Parminter, María de los Angeles Ferrera Fernandez, Rocio Rodriguez-Sánchez, Ana M. Wägner

**Affiliations:** 1 Unidad de Endocrinología Pediátrica, Complejo Hospitalario Universitario Insular Materno Infantil de Las Palmas de Gran Canaria, Las Palmas de Gran Canaria, Spain; 2 Asociación Canaria para la Investigación Pediátrica (ACIP canarias), Las Palmas, Spain; 3 Instituto Universitario de Investigaciones Biomédicas y Sanitarias de la Universidad de Las Palmas de Gran Canaria, Las Palmas de Gran Canaria, Spain; 4 Preventive Medicine and Public Health Area, University of La Laguna, Santa Cruz de Tenerife, Spain; 5 Institute of Biomedical Technologies, University of La Laguna, Santa Cruz de Tenerife, Spain; 6 Servicio de Inmunología, Complejo Hospitalario Universitario Insular Materno Infantil de Las Palmas de Gran Canaria, Las Palmas de Gran Canaria, Spain; 7 Servicio de Pediatría Complejo Hospitalario Universitario Insular Materno Infantil de Las Palmas de Gran Canaria, Las Palmas de Gran Canaria, Spain; 8 Genomics Division, Instituto Tecnológico y de Energías Renovables (ITER), Santa Cruz de Tenerife, Spain; 9 CIBER de Enfermedades Respiratorias (CIBERES), Instituto de Salud Carlos III, Madrid, Spain; 10 Facultad de Ciencias de la Salud, Universidad Fernando de Pessoa Canarias, Las Palmas de Gran Canaria, Spain; 11 Servicio de Endocrinología y Nutrición, Complejo Hospitalario Universitario Insular Materno Infantil de Las Palmas de Gran Canaria, Las Palmas de Gran Canaria, Spain

**Keywords:** Genetics, HLA, Pediatrics, Type 1 diabetes

## Abstract

**Abstract:**

The Canary Islands inhabitants, a recently admixed population with significant North African genetic influence, has the highest incidence of childhood-onset type 1 diabetes (T1D) in Spain and one of the highest in Europe. *HLA* accounts for half of the genetic risk of T1D.

**Aims:**

To characterize the classical *HLA-DRB1* and *HLA-DQB1* alleles in children from Gran Canaria with and without T1D.

**Methods:**

We analyzed classic *HLA-DRB1* and *HLA-DQB1* alleles in childhood-onset T1D patients (*n* = 309) and control children without T1D (*n* = 222) from the island of Gran Canaria. We also analyzed the presence or absence of aspartic acid at position 57 in the *HLA-DQB1* gene and arginine at position 52 in the *HLA-DQA1* gene. Genotyping of classical *HLA-DQB1* and *HLA-DRB1* alleles was performed at two-digit resolution using Luminex technology. The chi-square test (or Fisher's exact test) and odds ratio (OR) were computed to assess differences in allele and genotype frequencies between patients and controls. Logistic regression analysis was also used.

**Results:**

Mean age at diagnosis of T1D was 7.4 ± 3.6 years (46% female). Mean age of the controls was 7.6 ± 1.1 years (55% female). DRB1*03 (OR = 4.2; *p* = 2.13^–13^), DRB1*04 (OR = 6.6; *p* ≤ 2.00^–16^), DRB1* 07 (OR = *0.37; p* = 9.73^–06^), DRB1*11 (OR = *0.17; p* = 6.72^–09^), DRB1*12, DRB1*13 (OR = *0.38; p* = 1.21^–05^), DRB1*14 (OR = *0.0; p* = 0.0024), DRB1*15 (OR = *0.13; p* = 7.78^–07^) and DRB1*16 (OR = *0.21; p* = *0.00*3*)* exhibited significant differences in frequency between groups*.* Among the DQB1* alleles, DQB1*02 (OR: 2.3; *p* = 5.13^–06^), DQB1*03 (OR = 1.7; *p* = 1.89^–03^), DQB1*05 (OR = 0.64; *p* = 0.027) and DQB1*06 (OR = 0.19; *p* = 6.25^–14^) exhibited significant differences. A total of 58% of the studied *HLA-DQB1* genes in our control population lacked aspartic acid at position 57.

**Conclusions:**

In this population, the overall distributions of the *HLA-DRB1* and *HLA-DQB1* alleles are similar to those in other European populations. However, the frequency of the non-Asp-57 *HLA-DQB1* molecules is greater than that in other populations with a lower incidence of T1D. Based on genetic, historical and epidemiological data, we propose that a common genetic background might help explain the elevated pediatric T1D incidence in the Canary Islands, North-Africa and middle eastern countries.

**Supplementary Information:**

The online version contains supplementary material available at 10.1186/s12887-024-04983-w.

## Research in context

What is already known about this subject?

- The Canary Islands have the highest incidence to date of T1D in Spain.
- The HLA region accounts for up to 50% of the genetic risk for type 1 diabetes (T1D).
- The Canary Islands original inhabitants are genetically related to the current Berber population, and share a high incidence of pediatric T1D with north-African and middle eastern countries.

What is the key question?

- Is there a genetic basis to the increased T1D in the pediatric age group in the Canary Islands?

What are the new findings?

- There is a low prevalence of Asp57 HLA-DQB1 molecules in the population from Gran Canaria without T1D compared to other regions with a lower T1D incidence. Our findings support the seemingly protective role of HLA-DQB1 Asp57 molecules in T1D (especially HLA-DQB1*06).
- We hypothesize that a common genetic background, resulting from the migration of Arab and North African populations, and the resulting genetic admixture, may help explain the increased incidence of T1D in the pediatric age group in Arab, North African, Sardinian and Canarian populations.

How might this impact on clinical practice in the foreseeable future?

- A deeper understanding of the root causes of T1D in our population might prove beneficial once an individualized approach to the treatment of T1D becomes available.

## Introduction

Type 1 diabetes mellitus (T1D) is a multi-factorial disease resulting from autoimmune destruction of pancreatic β cells, limiting the body´s ability to produce insulin [1]. It is the most common type of diabetes in childhood and adolescence [2]. The development of T1D is influenced by a complex interplay between genetic factors and environmental exposures, which are not fully understood [3]. The Type 1 Diabetes Genetics Consortium (T1DGC) identified more than 75 different genetic risk regions for T1D [4], with the human leucocyte antigen (HLA) region accounting for 40–50% of that genetic susceptibility [5]. The HLA region comprises three major coding regions, and the proteins encoded by HLA class I and II are primarily responsible for binding and presenting antigens to T lymphocytes, playing a crucial role in the development of autoimmune diseases like T1D [6]. However, the influence of HLA in the appearance of T1D is not simple. Numerous studies have reported substantial ethnic differences in the risk and protective effects conferred by various HLA alleles and haplotype combinations. These findings underscore the importance of conducting population-specific characterizations of HLA associations with T1D to accurately assess disease risk and understand the underlying mechanisms.

The incidence of T1D varies worldwide, with the highest current incidence rates occurring in Northern Europe and Middle Eastern countries [7]. Incidence ranges from 52/100,000 in children < 15 years of age in Finland to 1–2/100,000 in Southeast Asia and the Western Pacific [7]. Most countries have experienced a global increase [8], and recent analyses predict even greater increases in the coming decades [9].

Located nearly 100 km west of Morocco, the Canary Islands archipelago is the southernmost region of Spain. With a T1D incidence in the pediatric age group of 30–32/100,000 for the last 15 years in Gran Canaria [10, 11] and the period 1993–2007 in the island of La Palma [12], it is the highest described to date in Spain [13] and one of the highest in Europe [14]. The current inhabitants are the result of a historical admixture of Western Europeans, North Africans, and Sub-Saharan Africans. The aboriginal population of the Canary Islands originally came from North Africa, giving its inhabitants a common ancestry with the current Berber population. In fact, genetic studies of the current population have shown that > 70% of the influences can be traced to Iberia (mainly Galicia and Portugal [15]), approximately 22% from the populations of Northwest Africa, and a small proportion is linked to sub-Saharan African influences (3%) [16]. These studies also confirmed that the last admixture event occurred approximately 14 generations ago, which is within the time frame of the Spanish conquest of the archipelago [16]. Furthermore, certain genomic regions of the current population have greater-than-expected African genetic ancestry and were shown to be significantly enriched in genes involved in diseases prevalent in the Canarian population, including diabetes. Specifically, one of these regions is *HLA* (16).

In accordance with the relative isolation of the population of the Canary Islands and the evident footprints of genetic inbreeding on some of the islands [16], a high prevalence of monogenic diseases, such as Wilson´s disease [17], familial hypercholesterolemia [18], congenital hyperinsulinism [19], and primary hyperoxaluria [20], among others, has been described in this population. Given the high incidence of T1D in both the Canary Islands [10] and North African countries [21] and the important recent genetic admixture with North African populations, we hypothesized that differences in the prevalence of high-risk or protective variants of HLA class II alleles between the Canary Islands and other populations could help explain the higher incidences in the pediatric age group.

## Methods

### Study design

This is a case‒control study with unrelated subjects. The Strengthening the Reporting of Observational Studies in Epidemiology (STROBE) guidelines [22] were followed in the reporting of the study.

### Setting

The study was conducted on the population from the island of Gran Canaria. Peripheral blood samples from patients with T1D were collected immediately after onset between 2010 and 2017 as part of the initial evaluation defined in our protocol for the diagnosis of T1D. In addition, peripheral blood samples were collected from healthy children between April and June 2022 as part of the protocol of a randomized, controlled trial on the effectiveness of an obesity prevention program [23] (clinical trial number 44205, www.aspredicted.org).

### Participants

This study was conducted according to the tenets of the Declaration of Helsinki after approval by the Ethics Committee of Hospital Universitario de Gran Canaria Dr. Negrín (protocol codes 2019–477-1 and 2020–356-1, approved on November 28th, 2019, and October 2nd, 2020, respectively). Informed consent was obtained from all subjects included in the study, as well as from their parents or guardians.

Patients were defined as having new-onset T1D before the age of 14 years. The American Diabetes Association (ADA) criteria were used for the diagnosis of T1D [24]. Controls were recruited from healthy schoolchildren aged 6–9 years. Peripheral blood samples were collected after parental and child consent were obtained from a sample of 13 schools on the island of Gran Canaria participating in an intervention study to evaluate the effectiveness of an obesity prevention program. The schools were selected with the approval of their school board. The number of children with T1D who participated in the study was defined by the number of onsets during the period 2010–2017. The number of children in the control group was defined by the number of children whose parents consented to their participation and blood sampling in the study.

### Anti-islet autoantibodies

T1D-related autoantibodies were measured at disease onset in our patients by radioimmunoassay (RIA) at Reference Laboratory S.A. (Barcelona, Spain). An islet antigen 2 (IA2) autoantibody RIA kit from RSR (Ldt, Cardiff, UK) with 125I-labeled IA2 was used for the detection of IA2 antibodies. Glutamic acid decarboxylase (GAD) autoantibody RIA kit from RSR with 125I-labeled GAD was used to detect GAD antibodies. The DIAsource AIA-100 kit was used for the detection of anti-insulin antibodies.

### DNA extraction and HLA genotyping

Peripheral blood samples were preserved in ethylenediaminetetraacetic acid (EDTA) tubes. Genomic DNA extraction method was an automated magnetic particle-based method using the commercial Maxwell DNA Purification Kit from Promega. Genotyping of the *HLA-DQB1* and *HLA-DRB1* alleles was performed with sequence-specific oligonucleotides (PCR-SSO) using Luminex technology. In these assays, oligonucleotide probes are attached to microspheres that are analyzed in blocks using a customized cytometer (Luminex) and specific software. Commercial LabType SSO Class II kits (One Lambda) were used to genotype the *HLA-DQB1* and *HLA-DRB1* loci. Characterization was performed up to a two-digit resolution in all patients. Estimation was performed up to the fourth digit in a subset of 75 children with T1D (those with T1D onset during a 3 year period in which our laboratory changed its protocol and performed HLA 4th digit estimation routinely) and all control participants. The frequency of *HLA-DQB1* molecules with aspartic acid at position 57 (Asp57; considered to be a protective molecule [25]) and *HLA-DQA1* with arginine at position 52 (Arg52; considered to be a risk molecule [26]) was assessed based on intermediate resolution typing and linkage disequilibrium with the *HLA-DRB1* locus. The *HLA-DQB1* molecules with Asp57 considered were *02:03, *03:01, *03:03, *04, *05:03, *06:01, *06:02, and *06:03. The *HLA-DQA1* molecules with Arg52 considered were *03:01, *04:01, and *05:01. A high-risk HLA genotype was defined by the presence of the HLA-DRB1*03 and/or HLA-DRB1*04 and/or HLA-DQB1*02 and/or HLA-DQB1*03 alleles [5].

### Statistical analysis

Descriptive statistics were used to calculate the frequency of HLA class II in the population. We examined the odds ratio (OR) and 95% confidence interval (95% CI) after comparing the frequency of each allele and genotype between study groups. The chi-square test (or Fisher's exact test when appropriate) and odds ratio (OR) were used to compare differences in allele and genotype frequencies between controls and T1D patients. To estimate the risk of T1D, logistic regression models were adjusted for sex, and were used to derive the ORs and 95% CIs. The significance threshold was set at *p* = 2.94E − 3 after Bonferroni correction based on the number of alleles tested considering both genes together.

Based on the identification of alleles associated with earlier disease onset, we investigated whether these effects varied with age at diagnosis to determine the threshold at which the allelic effects were most pronounced. To address this issue, we employed a sequential addition approach for the analysis of case‒control data, applicable in scenarios where a quantitative trait is measured solely in cases and where no defined threshold is known [27]. In summary, individual associations (for HLA-DRB1*03 and *04) were calculated using the same method as before but iteratively for each subset of cases formed by including patients with T1D at each incremental increase of 1 year in the age of diagnosis (from 1 to 15 years of age). The *P* value was subsequently obtained by multiple logistic regression adjusting by sex. These statistical analyses were performed using the statistical package SPSS v.29.0 (IBM Corp. Released 2022. IBM SPSS Statistics for Windows, Version 29.0. Armonk, NY: IBM Corp) and R v.3.6.3 [28].

## Results

### Participants

A total of 309 children diagnosed with T1D (mean age at diagnosis: 7.4 ± 3.7 years, 46% female) and 222 healthy controls (mean age: 7.6 ± 1.1 years, 55% female) were included in the study.

### Pancreatic autoantibodies

Overall, 90.4% of the T1D patients were positive for at least one autoantibody, the most common being anti-GAD (73%), followed by anti-IA2 (66%) and anti-insulin (28.7%). ZnT8 autoantibodies were not measured in our patients.

### *HLA-DRB1* associations

Among the *HLA-DRB1* alleles, the frequency of HLA-DRB1*03 and HLA-DRB1*04 is higher in patients than in controls (91% vs 42%; *p* < 0.00001), with HLA-DRB1*04 presenting the largest OR for T1D. HLA-DRB1* 07, *11, *13, *14, *15 and *16 had an increased frequency in the control group, with DRB1*13 being the most frequent*.* Regarding the genotype frequencies, HLA-DRB1*01/04, *03/03, *03/04 and *04/13 were significantly more frequent in patients, HLA-DRB1*03/03 presented the largest OR, and HLA-DRB1*03/04 was the most common genotype among T1D patients. HLA-DRB1*03/13, 07/11, 07/13, 07/15, 07/07, 11/13, 11/15, 13/13, and *15/15* were significantly more frequent in the controls (Table 1 and Supplementary Table 1).

**Table 1:** *HLA-DRB1* allele frequency in children with T1D and controls

Allele	Cases (2N)	Controls (2N)	OR	95%CI	*P*-value
	618	444			
*01	55	40	0.94	0.05—1.47	0.79
*03	199	52	4.27	2.89—6.28	2.13^–13^
*04	219	55	6.61	4.45—9.81	< 2.00^–16^
*07	42	72	0.37	0.24—0.57	9.73^–06^
*08	14	13	0.74	0.34—1.62	0.46
*09	7	7	0.73	0.25—2.12	0.56
*10	4	6	0.47	0.13—1.69	0.25
*11	17	55	0.17	0.09—0.31	6.72^–09^
*13	48	77	0.39	0.25—0.59	1.21^–05^
*14	0	7	0		0.0024^†^
*15	8	41	0.17	0.06—0.30	7.78^–07^
*16	5	15	0.21	0.07—0.60	3.50^–03^

Only alleles with ≥ 5 counts when considering cases and controls together were included in the table

*OR* Odds ratio, *95% CI* 95% Confidence Interval

^†^Fisher exact test

### *HLA-DQB1* associations

Regarding *HLA-DQB1*, 95.5% of cases carried HLA-DQB1*02 and/or *03, compared to 83.1% of controls (*p* < 0.00001). The protective allele HLA-DQB1*06 was present in 12.6% of cases (only 0.6% in homozygosis) and 41% of controls (5% in homozygosis) (*p* < 0.00001). HLA-DQB1*02 and HLA-DQB1*03 were more prevalent in cases, while HLA-DQB1*05 and HLA-DQB1*06 were more frequent in controls. Regarding genotypes, HLA-DQB1*02/02 and HLA-DQB1*02/03 were significantly more frequent in cases, while HLA-DQB1*02/06, *03/06, *05/06 and *06/06 were more frequent in controls (Table 2 and Supplementary Table 2).

**Table 2:** *HLA-DQB1* allele frequency in children with T1D and controls

Allele	Cases (2N)	Controls (2N)	OR	95%CI	*P*-value
	618	444			
*02	262	124	2.31	1.61—3.30	5.13^–06^
*03	232	132	1.75	1.23—2.49	1.89^–03^
*04	12	11	0.74	0.31—1.71	0.48
*05	71	69	0.64	0.43—0.95	0.028
*06	41	108	0.19	0.12—0.29	6.25^–14^

*OR* Odds ratio, *95% CI* 95% confidence interval

### Combinations of *HLA-DRB1* and *HLA-DQB1*

We also examined combinations of these alleles and found that certain types of alleles were more frequent in T1D patients than in controls. Regarding the *HLA-DRB1-DQB1* combinations, *01/04–03/05, *03/03–02/02, *03/04–02/02, and *03/04–02/03 were significantly more frequent in patients, whereas *01/11–03/05, *11/13–03/06, *11/15–03/06, *13/16–05/06, *15/15–06/06, *04/13–02/06, *07/11–02/03 and *07/13–02/06 were significantly more frequent in controls (Supplementary Table 3).

### Estimation up to the fourth digit of *HLA-DRB1* and *HLA-DQB1*

An estimation of a small subset of patients with T1D (75 patients [25%]) allowed us to characterize the DRB1* and DQB1* alleles up to the 4th digit resolution (Table 3). We were able to estimate up to the 4th digit in all control patients (Table 4). The only significant differences in 4-digit characterization were found for the DRB1* 04:03 (*p* = 0,025) (more frequent in controls), 04:05 (*p* = 0,023) (more frequent in T1D patients), 04:07 (*p* = 0,025) (more frequent in controls) and DQB1*02 alleles (with 02:01 being more frequently found in T1D patients, and 02:02 in controls (*p* = 0,000)). Estimations in patients with T1D and controls are shown in more detail in Supplementary Tables 4 and 5.

**Table 3:** Estimation of *DRB1* and *DQB1* up to the 4th digit in patients with T1D

Allele (N)	4th digit estimation (%)	4th digit estimation (%)	4th digit estimation (%)	4th digit estimation (%)
DRB1*01 (12)	01:01 (58)	01:02 (42)		
DRB1*03 (44)	03:01 (98)	03:05 (2)		
DRB1*04 (57)	04:01 (19)	04:02 (16)	04:04 (14)	04:05 (51)
DRB1*07 (6)	07:01 (100)			
DRB1*08 (3)	08:01 (67)	08:04 (33)		
DRB1*10 (2)	10:01			
DRB1*11 (3)	11:01 (67)	11:03 (33)		
DRB1*13 (13)	13:01 (46)	13:02 (31)	13:03 (23)	
DRB1*15 (2)	15:01			
DRB1*16 (1)	16:01			
DQB1*02 (57)	02:01 (86)	02:02 (14)		
DQB1*03 (61)	03:01 (13)	03:02 (85)	03:03 (2)	
DQB1*04 (1)	04:02			
DQB1*05 (13)	05:01 (92)	05:02 (8)		
DQB1*06 (12)	06:02 (8)	06:03 (47)	06:04 (39)

**Table 4:** Estimation of *DRB1* and *DQB1* up to the 4th digit in control children

Allele (N)	4th digit estimation (%)	4th digit estimation (%)	4th digit estimation (%)	4th digit estimation (%)	4th digit estimation (%)	4th digit estimation (%)	4th digit estimation (%)	4th digit estimation (%)
DRB1*01 (39)	01:01 (41)	01:02 (49)	01:03 (10)					
DRB1*03 (52)	03:01 (96)	03:02 (4)						
DRB1*04 (54)	04:01 (19)	04:02 (13)	04:03 (9)	04:04 (17)	04:05 (29)	04:06 (2)	04:07 (9)	04:10 (2)
DRB1*07 (72)	07:01 (100)							
DRB1*08 (13)	08:01 (61)	08:03 (23)	08:04 (8)	08:02 (8)				
DRB1*09 (7)	09:01 (100)							
DRB1*10 (6)	10:01 (100)							
DRB1*11 (55)	11:01 (59)	11:02 (16)	11:03 (9)	11:04 (16)				
DRB1*12 (4)	12:01 (100)							
DRB1*13 (77)	13:01 (49)	13:02 (35)	13:03 (12)	13:56 (1)	13:05 (1)	13:36 (1)	13:4 (1)	
DRB1*14 (7)	14:01 (100)							
DRB1*15 (42)	15:01 (88)		15:03 (12)					
DRB1*16 (14)	16:01 (86)	16:02 (14)						
DQB1*02 (123)	02:01 (46)	02:02 (54)						
DQB1*03 (132)	03:01 (54)	03:02 (30)	03:03 (10)	03:19 (6)				
DQB1*04 (11)	04:02 (100)							
DQB1*05 (70)	05:01 (69)	05:02 (23)	05:03 (8)					
DQB1*06 (106)	06:02 (34)	06:03 (39)	06:04 (21)	06:09 (4)	06:01 (2)			

As expected, the frequency of (1 or 2) *HLA-DRB1* risk alleles was greater in patients with T1D (OR = 1.5; *p* = 0.02; OR = 12.3; *p* < 0.00001, respectively). In the case of *HLA-DQB1* alleles, the presence of two risk alleles was more frequent in patients (OR = 3.5; *p* < 0.00001), while the presence of one risk allele or none was more frequent in controls (OR = 0.48; *p *= 0.00006 and OR = 0.19; *p *< 0.00001, respectively) (Supplementary Tables 6 and 7).

### Asp-57 and non-Asp-57 molecules

Focusing on the *HLA-DQB1* molecules with Asp57, we found evident differences in the frequency of homozygous and heterozygous combinations in the population of Gran Canaria (Table 5).

**Table 5:** Adapted from Dorman et al. [29]. Presence/absence of Asp57 in patients and controls in various population [30–36]

		T1D				Controls			T1D incidence
	N/N(%)	N/A(%)	A/A(%)	N(%)	N/N(%)	N/A(%)	A/A(%)	N(%)	
**Finland**(30)	**74**	**22**	**3**	**85**	**18**	**57**	**25**	**46**	**52.2/100,000**(7)
Sardinia	100	0	0	100	38	47	15	62	45/100,000(31)
Norway	80	16	4	89	27	51	22	53	32.7/100,000(32)
**Gran Canaria**	**78**	**19**	**2**	**88**	**35**	**46**	**19**	**58**	**30/100,000**(10,11)
US Caucasians	61	39	0	81	20	46	34	43	27.3/100,000(33)
US Blacks	73	27	0	87	18	37	45	36	20.8/100,000(33)
**France**(34)	**88**	**12**	**0**	**94**	**23**	**55**	**22**	**51**	**19.1/100,000**(35)
Chinese	6	72	22	42	0	8	92	4	3.1/100,000(36)
**Japan**(37)	**3**	**49**	**48**	**27**	**8**	**34**	**58**	**25**	**2.25/100,000**(38)
**Erlich et al.** [5] (Europe, North America, Australia/New Zealand)				**91**				**52**	

*N/N* non-Asp57 homozygosity, *N/A* non-Asp57 heterozygosity, *A/A* Asp57 homozygosity, *N* non-Asp57 gene frequency

Evaluating all the Asp57 molecules (HLA-DQB1*02:03, *03:01, *03:03, *04, *05:03, *06:01, *06:02, and *06:03), we found that *02:03 was very rare, while *03:01 and *03:03 represented only 15% of the HLA-DQB1*03 alleles in children with T1D (Table 3), and HLA-DQB1*04 and *05 were rare in both patients and controls (Tables 3 and Supplementary Tables 4 and 5). Thus, HLA-DQB1*06 (*06:01, *06:02, and *06:03) was the most common Asp57 molecule and carries most of the weight for the protective effect attributed to the Asp57 *HLA-DQB1* alleles in this population.

We performed a correlation analysis using the non-Asp57 gene frequencies published by Dorman et al. [29] and added the data from this study. The correlation between the absence of Asp57 molecules and T1D incidence was strong even after including data from Gran Canaria. The initial correlation analysis showed an *R *= 0.98, *p* = 0.003. The correlation was similar (*R* = 0.95; *p* = 0.003) when the data from this study were added. We also added data from other publications where the incidence did not seem to correlate so well with the absence of Asp57 genes (*R* = 0.76; *p* = 0.017) (Table 5).

### Arg52 and non-Arg52 molecules

When assessing *HLA-DQA1* molecules with Arg52, some combinations were evidently different between cases and controls (Table 6). We added the presence and combination of the *HLA-DQB1* Arg57 molecules to allow comparison with the findings of Khalil et al. [26]. The table represents all possible combinations of susceptible (S) and protective (P) *HLA-DQA1* and *HLA-DQB1* Arg52 and Asp57 molecules.

**Table 6:** Frequency of appearance of susceptible DQA1 and DQB1 alleles

Combinations	DQA1*	DQB1*	T1D (*N* = 69) n(%)	Controls (*N* = 222) n(%)
1	S	S	15 (21.7)	9 (4)
	S	S		
2	S	S	2 (2.9)	9 (4)
	S	P		
3	S	S	20 (29)	42 (18.9)
	P	S		
4	P	S	14 (20.3)	33 (14.8)
	S	P		
5	S	S		
	P	P		
6	P	S	11 (15.9)	30 (13.5)
	P	S		
7	S	P	0	0
	S	P		
8	P	S	4 (5.8)	61 (27.4)
	P	P		
9	P	P	2 (2.9)	8 (3.6)
	S	P		
10	P	P	1 (1.5)	30 (13.5)
	P	P		

*S* Susceptible chain (DQB1*Asp57 negative; DQA1*Arg52 positive)

*P* Protective chain (DQB1*Asp57 positive; DQA1*Arg52 negative)

*T1D* type 1 diabetes

### Age effect

**Fig. 1:**
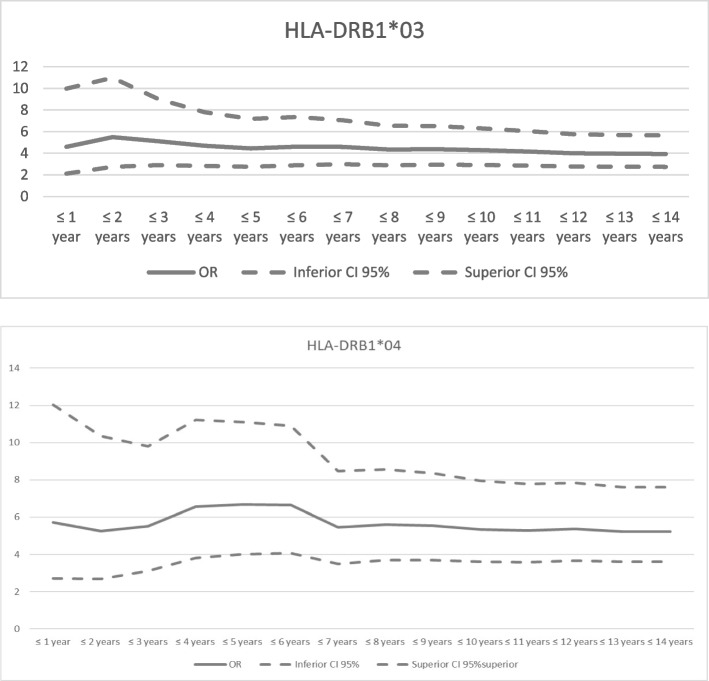
Effects of HLA-DRB1*03 and *04 according to age at T1D diagnosis

We analyzed the effect of the highest risk *HLA-DRB1* alleles on the age of T1D onset. By analyzing the risk-documented alleles (HLA-DRB1*03 and *04), we observed that the effects were greatest for 2 years of age at diagnosis for HLA-DRB1*03 (OR, 5.49; 95% CI, 2.75–10.98; *P* < 0.001) and for 5 years at diagnosis for HLA-DRB1*04 (OR, 6.67; 95% CI, 4.01–11.09; *P* < 0.001) (Fig. 1).

When comparing the presence of risk alleles among different age groups, we did not find significant differences. However, a trend was found for the presence of HLA-DRB1*04 among children aged 1–5 years (Table 7).

**Table 7:** Probability of presenting HLA-DRB1*03 and *04 in each age group (at diagnosis)

	1–5 years of age	6–10 years of age	11–14 years of age
HLA-DRB1*03			
OR	1.20	1.12	0.71
95%CI	0.74–1.94	0.71–1.77	0.42–1.18
*P* value	0.47	0.63	0.19
HLA-DRB1*04			
OR	1.67	0.71	0.88
95%CI	0.99–2.83	0.44–1.14	0.52–1.50
*P* value	0.054	0.15	0.64

## Discussion

This study investigated the genetic characteristics of T1D patients from the island of Gran Canaria and young children without T1D from the same island. In general, the HLA profile is similar to that described for other populations with childhood-onset T1D. We found small differences, with similar frequencies of HLA-DRB1*03 and *04 in our population, while HLA-DRB1*03 was found more frequently in T1D children from the rest of Spain. Additionally, for *HLA-DQB1* we found a lower prevalence of protective molecules (Asp57-positive) in the control group compared to other non-diabetic populations with a lower incidence of T1D.

As the genetic region associated with the highest risk for T1D, class II *HLA* has been extensively studied. Genetic differences have been described between European, African, and Asian populations [5, 37, 38] (Supplementary Table 8). A lower frequency of HLA-DRB1*03 and *04 [37, 39], variable presence of *HLA-DQB1* Asp57 [29] and *HLA-DQA1* Arg52 molecules [26], and other genetic variation have been used to explain the differences in incidence between populations.

### Risk alleles for T1D

In agreement with the findings of previous reports, our study revealed a predominance of HLA-DRB1*03 and *04, as well as HLA-DQB1*02 and *03, in patients with T1D, with a non-significant trend suggesting a higher effect for the HLA-DRB1*04 allele peaking among children aged 1–5 years. Previous studies in populations from the Canary Islands and other populations from mainland Spain showed that patients diagnosed with T1D have a greater proportion of *HLA-DRB1* risk alleles than healthy controls. A previous study by our group [40] evaluated the results of Spanish participants in the T1DGC (142 families, 49 from the Canary Islands), comparing affected and unaffected family members from the Canary Islands with those from the rest of Spain. The study concluded that the high incidence of childhood-onset T1D in the Canarian population does not seem to be explained by a higher prevalence of high-risk class II HLA haplotypes in families with this disease. Our results here point in the same direction, with the healthy control group having HLA risk profiles similar to those described in other populations with a lower incidence of T1D. Santana et al. also evaluated the prevalence of *DRB1* risk alleles and reported a higher frequency of HLA-DRB1*03 than of HLA-DRB1*04 in mainland Spain, whereas in the Canary Islands population, the frequency of both alleles was similar. The present study also revealed a similar prevalence of both risk alleles in the Canarian population.

Regarding studies in other Spanish populations, Urrutia et al. [41] compared the number of *HLA-DRB1* risk alleles between 160 T1D patients, 74 patients with monogenic diabetes, and 75 healthy controls. They reported that 48% and 44.3% of pediatric patients with T1D had 2 and 1 *HLA-DRB1* risk alleles, respectively, which was significantly greater than the proportion found in patients with monogenic diabetes and healthy controls. Our results in T1D patients are very similar (45% and 46%, respectively).

When compared to Europeans [5] and North Africans [42–44], the *HLA-DRB1* alleles in the T1D patients and healthy individuals were similar to those described by other studies. We present differences with African and Asian populations (particularly Chinese and Japanese), were DRB1*09:01 is one of the main risk alleles (supplementary Table 8). The similitudes with European populations remain when comparing results after high-resolution estimation (of up to the fourth digit in patients with T1D). HLA-DRB1*03:01 was the main *03 allele (98%), while HLA-DRB1*04:05 (51%) was the main *04 allele in the T1D population. Compared to our control group, there were small differences in the 4-digit characterization, with only DRB1*04 and DQB1*02 presenting differences between both groups (DRB1*04:03, DRB1*04:05 and DRB1*04:07 were more frequent in controls while DRB1*04:05 in T1D patients, and DQB1*02:01 being more frequently found in T1D patients, while DQB1*02:02 in controls).

### Protective alleles

In control children, DRB*13 was the most common allele, with DRB*15 presenting the lowest OR. Among the *HLA-DQB1* alleles, HLA-DQB1*02 and *03 were also the most common, followed by HLA-DQB1*06, which had the lowest OR. These results are, in general, similar to those reported by other authors (Supplementary Table 8).

Of particular interest is the presence/absence of aspartic acid at position 57 in *HLA-DQB1* molecules. Several studies, some dating back to 1987 [45], associated the absence of the amino acid aspartic acid at residue 57 of the HLA-DQB1 chain (Asp57-) with T1D susceptibility [29, 46]. The presence of Asp57 appears to be associated with dominant protection [25]. The mechanisms underlying its effect on T1D susceptibility could be related to how it affects the binding affinity of the HLA molecule with T cells and its effects on thymic selection and T-cell receptor responsiveness in the periphery [47]. In 1990, Dorman et al. [29] reported an inverse correlation between the presence of Asp57 and the incidence of T1D in different populations (Sardinia, Norway, the U.S. Caucasian and African American populations and China). In 2000, another report [48] compared the frequency of alleles in European, African American, and Asian populations from the DiaMond Molecular Epidemiology Project and the 12th International Histocompatibility Workshop and Conference (aggregating data from 20 European populations worldwide). They focused on the HLA-DQB1*06 allele (the most common Asp57 molecule) and found a greater frequency in controls in all ethnic groups. This difference was more pronounced in controls from populations with the lowest incidence of T1D, African Americans and Asians (32% and 33% vs. 23%, respectively). Our findings are in good agreement with these studies (Table 4), as the low prevalence of *HLA-DQB1* Asp57 could partially explain the high incidence of T1D in the Canarian population. It is worth mentioning that published results regarding the possible effects of Asp57 molecules do not fully explain the variability in incidence rates (Table 4). Reijonen et al. [49] studied the presence of *HLA-DQB1* Asp57 molecules in the Finnish population. When comparing its prevalence in the general population with that in other countries, Asp57 molecules did not explain the variation in incidence rates. In 2001, Ronningen et al. investigated the relationship between HLA genotypes and the incidence of T1D in Europe [50]. While they reported the existence of a clear correlation between the incidence of T1D and HLA genes, most of the effects were attributed to the *HLA-DQ2*/*DQ8* (DQB1*02:01/DQB1*03:02) and *HLA-DQ4*/*DQ8* (DQB1*04:02/DQB1*03:02) genotypes. They found no correlation between T1D incidence and the population prevalence of genotypes without Asp57. A possible explanation for these differences could be the effect of other HLA and non-HLA genes, as well as environmental factors, on the development of T1D. Khalil et al. in 1990 investigated the effect of HLA genes on the occurrence of T1D in France [26]. They studied the effect of *HLA-DQA1* alleles and reported a clear relationship between the presence of Arg52 molecules in combination with Asp57- molecules and the risk of T1D. Studying 50 patients with T1D and 73 healthy controls, they found that only individuals with the Arg52/Asp57- molecules (both risk molecules) developed T1D. None of the individuals negative for Arg52 but with Asp57 molecules (both protective molecules) developed T1D. In the population of Gran Canaria, we found controls in which Arg52 was absent but Asp57 was present (both protective) (Table 5), whereas Khalil et al. did not. However, children with Arg52 but not Asp57 (both susceptible molecules) were much more likely to have T1D (73.9% vs 41.7% of controls), whereas children with Arg52- but with Asp57 were more likely to be healthy (26.1% T1D vs 58% of controls). These results reflect the importance of HLA alleles other than *HLA-DQB1* and *HLA-DRB1* in the susceptibility to T1D. The results published by Aydemir et al. [51] help to reinforce their importance by describing a haplotype of three variants in *HLA-DQA1* as modulators of T1D risk in children homozygous for *HLA-DR3*.

In other studies in Spanish populations, Escribano et al. [52] reported a greater frequency of HLA-DQB1*06 in Cantabrian controls than in patients with T1D. However, we were unable to make a direct comparison between our data and those of Dorman et al. with the information provided in their report. When comparing data from the Canary Islands and the rest of Spain, Santana et al. [40] found no difference between controls from the Canary Islands and the rest of the country. Nevertheless, they reported a similar prevalence of HLA-DQB1*06 in control subjects from the Canary Islands (6.4% vs 8.6%).

Regarding other protective alleles more frequently found in the control population, our results are similar to those reported by other authors, with the exception of HLA-DRB1*16. This allele had an OR of 0.24 (*p* < 0.05) in our sample, while it did not reach significance in Erlich et al.´s report [5] or other studies in different populations.

### African influence on the Canary Islands

In 2018, Guillen-Guio et al. [16] published the results of a study analyzing the European and African genomic influence in the current population of the Canary Islands. Based on SNP array data and whole-genome sequencing, they concluded that up to 34% of the Canarian genome is of recent African descent, with the predominance of African alleles in some chromosomal regions (where 40–50% of the genome is of African descent), which include HLA. These genetic findings reflect the importance of past migration from Arab and North African populations to their Western European neighbors and the resulting genetic admixture [53]. We hypothesize that this common genetic background may help explain the increased incidence of T1D in the pediatric age group in Arab, North African, and Canarian populations. Note that Kuwait, Qatar, Algeria, and Saudi Arabia are among the eight countries with the highest incidence of pediatric T1D worldwide, with values of 41.7, 38.1, 34.8 and 31.4/100,000, respectively [7, 10]. Furthermore, the incidence of T1D in Spain shows an increasing North–South gradient, with the highest incidence rates found in Andalusia [54]. Additionally, the Italian island of Sardinia has the highest incidence of pediatric T1D in Europe after Finland (45/100,000) [55]. Thus, the presence of alleles of recent African ancestry in southern Europe may play a role in the increased incidence of T1D in these regions [56].

Some of the strengths of our study are the number of subjects included (the greatest to date in our region) and the use of a control group without T1D. We also acknowledge that this study has several limitations. The limited sample size (when compared to multicenter or collaborative projects) and young age of our control group (some of whom could develop T1D in the future) are some of our limitations. Our HLA analysis is limited to two-digit resolution. Four-digit characterization is important since it is sometimes the only way to differentiate between risk and protective alleles (e.g., HLA-DQB1*03:02 is considered a risk allele, whereas *03:01 is considered neutral or protective [5]). Direct comparisons between studies are often difficult due to methodological differences in the analysis and the reporting of the results. Some authors describe the allelic characteristics of their population, while others describe the genotype. Our findings regarding the *HLA-DQB1* Asp57 molecules support an association with T1D incidence. However, it is important to bear in mind that this does not imply causality.

## Conclusion

The risk and protective alleles in patients with T1D and in the healthy population from the Canary Islands are similar to those described in mainland Spain and other European populations. There is a low prevalence of Asp57 *HLA-DQB1* molecules in the population from Gran Canaria without T1D compared to other regions with a lower T1D incidence. Our findings support the seemingly protective role of *HLA-DQB1* Asp57 molecules in T1D (especially HLA-DQB1*06), and their absence or presence may help to explain incidence variation across populations. Larger-scale and more comprehensive studies are needed to confirm the role of these genes in the pathogenesis of T1D. Historical and T1D epidemiologic data and genetic evidence also support the relationships among Arab, North African, southern Spanish, and Canarian populations.

## Supplementary Information

Supplementary Material 1.

## Data Availability

The dataset(s) supporting the conclusions of this article are included within the article (and its additional file(s)).
