# Supplementary material for: Does HLA explain the high incidence of childhood-onset type 1 diabetes in the Canary Islands? The role of Asp57 DQB1 molecules
Source: BMC Pediatr. 2024 Sep 6;24:569. doi: 10.1186/s12887-024-04983-w (PMC11378579; doi:10.1186/s12887-024-04983-w)
Supplement: Supplementary file 1 — Supplementary Material 1. [file 12887_2024_4983_MOESM1_ESM.docx]

**Supplementary tables**

Supplementary Table 1, *DRB1** genotypes

| Genotype | Cases | Controls | OR | 95%CI | Chi-square | P-value |
| --- | --- | --- | --- | --- | --- | --- |
| 01/13 | 5 | 6 | 0.59 | 0.18-1.97 | 0.7 | 0.38 |
| 01/03 | 16 | 5 | 2.4 | 0.86-6.57 | 2.9 | 0.08 |
| 01/04 | 24 | 6 | 3 | 1.22-7.55 |  | 0.013 ^†^ |
| 01/07 | 3 | 8 | 0.26 | 0.07-1 |  | 0.059 ^†^ |
| 11/13 | 0 | 9 |  |  |  | 0.0004 ^†^ |
| 11/15 | 0 | 6 |  |  |  | 0.0051 ^†^ |
| 13/13 | 2 | 8 | 0.17 | 0.04-0.83 |  | 0.002 ^†^ |
| 13/16 | 0 | 6 |  |  |  | 0.0051^†^ |
| 15/15 | 0 | 5 |  |  |  | 0.0124^†^ |
| 03/11 | 3 | 8 | 0.26 | 0.07-1 |  | 0.0592^†^ |
| 03/13 | 7 | 15 | 0.3 | 0.13-0.84 | 6.5 | 0.01 |
| 03/03 | 27 | 1 | 21.16 | 2.8-156.9 |  | <0.00001^†^ |
| 03/04 | 95 | 8 | 12.4 | 5.9-26.2 | 61.5 | 0.00001 |
| 03/07 | 10 | 7 | 1 | 0.36-2.5 | 0.59 | 0.4 |
| 04/11 | 9 | 4 | 1.5 | 0.47-5 |  | 0.57^†^ |
| 04/13 | 30 | 8 | 2.9 | 1.2-6.4 | 7.2 | 0.007 |
| 04/04 | 16 | 5 | 2.3 | 0.8-6.5 | 2.9 | 0.08 |
| 04/07 | 19 | 6 | 2.3 | 0.93-6 | 3.4 | 0.06 |
| 07/11 | 2 | 10 | 0.14 | 0.03-0.64 |  | 0.005^†^ |
| 07/13 | 3 | 15 | 0.14 | 0.04-0.47 |  | 0.0004^†^ |
| 07/15 | 0 | 6 |  |  |  | 0.0051^†^ |
| 07/07 | 0 | 6 |  |  |  | 0.0051^†^ |

^Only genotypes with ≥ 5 cases+controls were included in the table, † Fisher exact test^

Supplementary table 2, *DQB1** genotypes

| Genotype | Cases | Controls | OR | 95%CI | Chi-square | p-value |
| --- | --- | --- | --- | --- | --- | --- |
| 02/02 | 49 | 15 | 2.55 | 1.39-4.69 | 36 | 0.00001 |
| 02/03 | 116 | 39 | 2.77 | 1.8-4.2 | 23.9 | 0.00001 |
| 02/04 | 8 | 2 | 2.87 | 0.6-13.6 |  | 0.2^†^ |
| 02/05 | 28 | 22 | 1.39 | 0.77-2.5 | 0.1 | 0.74 |
| 02/06 | 12 | 29 | 0.26 | 0.13-0.53 | 15.8 | 0.00007 |
| 03/03 | 31 | 17 | 1.3 | 0.7-2.4 | 0.34 | 0.34 |
| 03/04 | 2 | 5 | 0.28 | 0.05-1.47 |  | 0.13^†^ |
| 03/05 | 31 | 22 | 0.99 | 0.56-1.77 | 0.002 | 0.96 |
| 03/06 | 21 | 29 | 0.48 | 0.26-0.86 | 36 | 0.00001 |
| 05/06 | 4 | 18 | 0.15 | 0.05-0.44 |  | 0.0001^†^ |
| 06/06 | 2 | 12 | 0.11 | 0.02-0.5 |  | 0.0013^†^ |

^Only genotypes with ≥ 5 cases+controls were included in the table,^ † ^Fisher exact test^

Supplementary Table 3, Genotype frequencies

| Genotype | Cases | Controls | OR | 95%CI | Chi-square | P-value |
| --- | --- | --- | --- | --- | --- | --- |
| 01/11-03/05 | 0 | 5 |  |  |  | 0.0124^†^ |
| 01/13-03/05 | 1 | 5 | 0.14 | 0.02-1.2 |  | 0.087^†^ |
| 01/15-05/06 | 0 | 6 |  |  |  | 0.0051^†^ |
| 01/03-02/05 | 16 | 5 | 2.3 | 0.86-6.5 | 2.9 | 0.08 |
| 01/04-03/05 | 20 | 6 | 2.49 | 0.98-6.3 | 3.9 | 0.047 |
| 01/07-02/05 | 3 | 8 | 0.26 | 0.07-1 |  | 0.059^†^ |
| 11/13-03/06 | 0 | 5 | 0 |  |  | 0.0124^†^ |
| 11/15-03/06 | 0 | 5 | 0 |  |  | 0.0124^†^ |
| 13/13-06/06 | 2 | 7 | 0.2 | 0.04-0.97 |  | 0.038^†^ |
| 13/16-05/06 | 0 | 5 | 0 |  |  | 0.0124^†^ |
| 15/15-06/06 | 0 | 5 | 0 |  |  | 0.0124^†^ |
| 03/03-02/02 | 27 | 1 | 20.79 | 2.8-154 |  | <0.00001^†^ |
| 03/04-02/02 | 7 | 1 | 5.1 | 0.63-41.9 |  | <0.00001^†^ |
| 03/04-02/03 | 88 | 7 | 12.2 | 5.5-27 | 56.4 | <0.00001 |
| 04/11-03/03 | 7 | 3 | 1.69 | 0.4-6.6 |  | 0.5^†^ |
| 04/13-02/06 | 1 | 8 | 0.09 | 0.01-0.7 |  | 0.005^†^ |
| 04/13-03/06 | 19 | 8 | 1.75 | 0.75-4 | 1.7 | 0.18 |
| 04/04-03/03 | 15 | 4 | 2.78 | 0.9-8.4 |  | 0.09^†^ |
| 04/07-02/03 | 16 | 4 | 2.98 | 0.98-9 |  | 0.06^†^ |
| 07/11-02/03 | 3 | 9 | 0.23 | 0.06-0.87 |  | 0.003^†^ |
| 07/13-02/06 | 1 | 9 | 0.08 | 0.01-0.6 |  | 0.0022^†^ |

^Only haplotypes with ≥ 5 cases+controls were included in the table, † Fisher exact test^

Supplementary Table 4, Low resolution HLA analysis and estimation of fourth digit in control children,

| **Low resolution *DRB1*** | **Low resolution *DQB1*** | **Estimated *DRB1*** | **Estimated *DQB1*** | **Estimated *DQA1*** |
| --- | --- | --- | --- | --- |
| DRB1*07, DRB1*09 | DQB1*02, DQB1*03 (DQ9) | DRB1*07:01, DRB1*09:01 | DQB1*02:02, DQB1*03:03 | DQA1*02:01, DQA1*03:01 |
| DRB1*03, DRB1*15 | DQB1*02, DQB1*06 | DRB1*03:01, DRB1*15:01 | DQB1*02:01, DQB1*06:02 | DQA1*01:02, DQA1*05:01 |
| DRB1*13, DRB1*-- | DQB1*06, DQB1*-- | DRB1*13:01, DRB1*13:02 | DQB1*06:03, DQB1*06:04 | DQA1*01:02, DQA1*01:03 |
| DRB1*03, DRB1*13 | DQB1*02, DQB1*06 | DRB1*03:01, DRB1*13:02 | DQB1*02:01, DQB1*06:04 | DQA1*01:02, DQA1*05:01 |
| DRB1*01, DRB1*04 | DQB1*03 (DQ7), DQB1*05 | DRB1*01:01, DRB1*04:01 | DQB1*03:01, DQB1*05:01 | DQA1*01:01, DQA1*03:01 |
| DRB1*07, DRB1*-- | DQB1*02, DQB1*-- | DRB1*07:01, — | DQB1*02:02, DQB1*02:02 | DQA1*02:02, DQA1*02:01 |
| DRB1*07, DRB1*11 | DQB1*02, DQB1*03 (DQ7) | DRB1*07:01, DRB1*11:01 | DQB1*02:02, DQB1*03:01 | DQA1*02:02, DQA1*05:05 |
| DRB1*07, DRB1*11 | DQB1*02, DQB1*03 (DQ7) | DRB1*07:01, DRB1*11:01 | DQB1*02:02, DQB1*03:01 | DQA1*02:01, DQA1*05:05 |
| DRB1*03, DRB1*04 | DQB1*02, DQB1*03 (DQ8) | DRB1*03:01, DRB1*04:04 | DQB1*02:01, DQB1*03:02 | DQA1*01:02, DQA1*05:01 |
| DRB1*01, DRB1*07 | DQB1*02, DQB1*05 | DRB1*01:01, DRB1*07:01 | DQB1*02:02, DQB1*05:01 | DQA1*01:01, DQA1*02:01 |
| DRB1*04, DRB1*13 | DQB1*03 (DQ8), DQB1*06 | DRB1*04:03, DRB1*13:02 | DQB1*03:02, DQB1*06:04 | DQA1*01:02, DQA1*03:01 |
| DRB1*03, DRB1*13 | DQB1*02, DQB1*03 (DQ7) | DRB1*03:01, DRB1*13:03 | DQB1*02:01, DQB1*03:01 | DQA1*05:01, ----------------- |
| DRB1*04, DRB1*13 | DQB1*02, DQB1*06 | DRB1*13:01, DRB1*04:04 | DQB1*02:01, DQB1*06:03 | DQA1*01:03, DQA1*03:01 |
| DRB1*04, DRB1*14 | DQB1*03 (DQ8), DQB1*05 | DRB1*04:05, DRB1*14:01 | DQB1*03:02, DQB1*05:03 | DQA1*01:01, DQA1*03:01 |
| DRB1*11, DRB1*15 | DQB1*03 (DQ7), DQB1*06 | DRB1*11:01, DRB1*15:01 | DQB1*03:01, DQB1*06:02 | DQA1*01:02, DQA1*05:05 |
| DRB1*11, DRB1*15 | DQB1*03 (DQ7), DQB1*06 | DRB1*11:02, DRB1*15:01 | DQB1*03:19, DQB1*06:02 | DQA1*01:02, DQA1*05:05 |
| DRB1*03, DRB1*13 | DQB1*02, DQB1*06 | DRB1*03:01, DRB1*13:01 | DQB1*02:01, DQB1*06:03 | DQA1*01:03, DQA1*05:01 |
| DRB1*08, DRB1*15 | DQB1*04, DQB1*06 | DRB1*08:01, DRB1*15:01 | DQB1*04:02, DQB1*06:02 | DQA1*01:02, DQA1*04:01 |
| DRB1*04, DRB1*07 | DQB1*02, DQB1*-- | DRB1*04:02, DRB1*07:01 | DQB1*02:01, DQB1*02:02 | DQA1*02:01, DQA1*03:01 |
| DRB1*07, DRB1*13 | DQB1*02, DQB1*06 | DRB1*07:01, DRB1*13:02 | DQB1*02:02, DQB1*06:04 | DQA1*01:02, DQA1*02:01 |
| DRB1*07, DRB1*11 | DQB1*02, DQB1*03 | DRB1*07:01, DRB1*11:01 | DQB1*02:02, DQB1*03:01 | DQA1*02:01, DQA1*05:05 |
| DRB1*03, DRB1*13 | DQB1*02, DQB1*03 (DQ7) | DRB1*03:01, DRB1*13:03 | DQB1*02:01, DQB1*03:01 | DQA1*05:01, -- |
| DRB1*07, DRB1*13 | DQB1*03 (DQ9), DQB1*06 | DRB1*07:01, DRB1*13:01 | DQB1*03:03, DQB1*06:03 | DQA1*01:03, DQA1*02:01 |
| DRB1*07, DRB1*15 | DQB1*02, DQB1*06 | DRB1*07:01, DRB1*15:01 | DQB1*02:02, DQB1*06:02 | DQA1*01:02, DQA1*02:01 |
| DRB1*07, DRB1*13 | DQB1*03 (DQ9), DQB1*06 | DRB1*07:01, DRB1*13:01 | DQB1*03:03, DQB1*06:03 | DQA1*01:03, DQA1*02:01 |
| DRB1*04, DRB1*07 | DQB1*02, DQB1*-- | DRB1*04:01, DRB1*07:01 | DQB1*02:01, DQB1*02:02 | DQA1*02:01, DQA1*03:01 |
| DRB1*01, DRB1*03 | DQB1*02, DQB1*05 | DRB1*01:01, DRB1*03:01 | DQB1*02:01, DQB1*05:01 | DQA1*01:01, DQA1*05:01 |
| DRB1*04, DRB1*15 | DQB1*03 (DQ8), DQB1*06 | DRB1*04:03, DRB1*15:03 | DQB1*03:02, DQB1*06:02 | DQA1*01:02, DQA1*03:01 |
| DRB1*07, DRB1*13 | DQB1*02, DQB1*06 | DRB1*07:01, DRB1*13:01 | DQB1*02:02, DQB1*06:03 | DQA1*01:03, DQA1*02:01 |
| DRB1*13, DRB1*-- | DQB1*03 (DQ7), DQB1*06 | DRB1*13:02, DRB1*13:03 | DQB1*03:01, DQB1*06:03 | DQA1*01:03, DQA1*05:05 |
| DRB1*01, DRB1*03 | DQB1*02, DQB1*05 | DRB1*01:01, DRB1*03:01 | DQB1*02:01, DQB1*05:01 | DQA1*01:01, DQA1*05:01 |
| DRB1*03, DRB1*11 | DQB1*02, DQB1*03 (DQ7) | DRB1*03:01, DRB1*11:02 | DQB1*02:01, DQB1*03:19 | DQA1*05:01, ---------------- |
| DRB1*03, DRB1*07 | DQB1*02, DQB1*-- | DRB1*03:01, DRB1*07:01 | DQB1*02:01, DQB1*02:02 | DQA1*02:01, DQA1*05:01 |
| DRB1*04, DRB1*10 | DQB1*03 (DQ7), DQB1*05 | DRB1*04:01, DRB1*10:01 | DQB1*03:01, DQB1*05:01 | DQA1*01:01, DQA1*03:01 |
| DRB1*04, DRB1*11 | DQB1*03 (DQ7), DQB1*06 | DRB1*04:07, DRB1*11:03 | DQB1*03:01, DQB1*06:03 | DQA1*01:03, DQA1*05:05 |
| DRB1*04, DRB1*15 | DQB1*03 (DQ7), DQB1*06 | DRB1*04:07, DRB1*15:01 | DQB1*03:01, DQB1*06:02 | DQA1*01:02, DQA1*03:01 |
| DRB1*01, DRB1*11 | DQB1*03 (DQ7), DQB1*05 | DRB1*01:02, DRB1*11:03 | DQB1*03:01, DQB1*05:01 | DQA1*01:01, DQA1*05:05 |
| DRB1*03, DRB1*07 | DQB1*02, DQB1*-- | DRB1*03:01, DRB1*07:01 | DQB1*02:01, DQB1*02:02 | DQA1*02:01, DQA1*05:01 |
| DRB1*11, DRB1*13 | DQB1*03 (DQ7), DQB1*-- | DRB1*11:02, DRB1*13:03 | DQB1*03:01, DQB1*03:19 | DQA1*05:05, -- |
| DRB1*07, DRB1*11 | DQB1*02, DQB1*03 (DQ7) | DRB1*07:01, DRB1*11:01 | DQB1*02:02, DQB1*03:01 | DQA1*02:01, DQA1*05:05 |
| DRB1*07, DRB1*11 | DQB1*02, DQB1*03 (DQ7) | DRB1*07:01, DRB1*11:02 | DQB1*02:02, DQB1*03:19 | DQA1*02:01, DQA1*05:05 |
| DRB1*07, DRB1*13 | DQB1*02, DQB1*06 | DRB1*07:01, DRB1*13:02 | DQB1*02:02, DQB1*06:04 | DQA1*01:02, DQA1*02:01 |
| DRB1*11, DRB1*13 | DQB1*03 (DQ7), DQB1*06 | DRB1*11:01, DRB1*13:01 | DQB1*03:01, DQB1*06:03 | DQA1*01:03, DQA1*05:05 |
| DRB1*04, DRB1*08 | DQB1*03 (DQ8), DQB1*04 | DRB1*04:04, DRB1*08:01 | DQB1*03:02, DQB1*04:02 | DQA1*03:01, DQA1*04:01 |
| DRB1*07, DRB1*13 | DQB1*03 (DQ9), DQB1*06 | DRB1*07:01, DRB1*13:02 | DQB1*03:03, DQB1*06:09 | DQA1*01:02, DQA1*02:01 |
| DRB1*01, DRB1*04 | DQB1*03 (DQ8), DQB1*05 | DRB1*01:02, DRB1*04:03 | DQB1*03:02, DQB1*05:01 | DQA1*01:01, DQA1*03:01 |
| DRB1*04, DRB1*13 | DQB1*03 (DQ8), DQB1*06 | DRB1*04:07, DRB1*13:02 | DQB1*03:02, DQB1*06:04 | DQA1*01:02, DQA1*03:01 |
| DRB1*13, DRB1*15 | DQB1*06, DQB1*-- | DRB1*13:02, DRB1*15:01 | DQB1*06:02, DQB1*06:04 | DQA1*01:02,-- |
| DRB1*03, DRB1*13 | DQB1*02, DQB1*06 | DRB1*03:01, DRB1*13:02 | DQB1*02:01, DQB1*06:04 | DQA1*01:02, DQA1*05:01 |
| DRB1*04, DRB1*-- | DQB1*03 (DQ8), DQB1*-- | DRB1*04:05, – | DQB1*03:02, –------------ | DQA1*03:01,------------------ |
| DRB1*11, DRB1*15 | DQB1*03 (DQ7), DQB1*05 | DRB1*11:01, DRB1*15:01 | DQB1*03:01, DQB1*05:02 | DQA1*01:02, DQA1*05:05 |
| DRB1*01, DRB1*11 | DQB1*03 (DQ7), DQB1*05 | DRB1*01:02, DRB1*11:01 | DQB1*03:01, DQB1*05:01 | DQA1*01:01, DQA1*05:05 |
| DRB1*13, DRB1*-- | DQB1*06, DQB1*-- | DRB1*13:01, – | DQB1*06:03, –---------------- | DQA1*01:02, DQA1*01:03 |
| DRB1*04, DRB1*10 | DQB1*03 (DQ8), DQB1*05 | DRB1*04:02, DRB1*10:01 | DQB1*03:02, DQB1*05:01 | DQA1*01:01, DQA1*03:01 |
| DRB1*07, DRB1*-- | DQB1*02, DQB1*-- | DRB1*07:01, — | DQB1*02:02, DQB1*02:02 | DQA1*02:01,-- |
| DRB1*07, DRB1*15 | DQB1*02, DQB1*06 | DRB1*07:01, DRB1*15:01 | DQB1*02:02, DQB1*06:02 | DQA1*01:02, DQA1*02:01 |
| DRB1*03, DRB1*-- | DQB1*02, DQB1*-- | DRB1*03:01, — | DQB1*02:01, –---------------- | DQA1*05:01, ---------------- |
| DRB1*11, DRB1*16 | DQB1*03 (DQ7), DQB1*06 | DRB1*11:01, DRB1*15:01 | DQB1*03:01, DQB1*06:02 | DQA1*01:02, DQA1*05:05 |
| DRB1*13, DRB1*16 | DQB1*05, DQB1*06 | DRB1*13:01, DRB1*16:01 | DQB1*05:02, DQB1*06:03 | DQA1*01:02, DQA1*01:03 |
| DRB1*15, DRB1*-- | DQB1*06, DQB1*-- | DRB1*15:01, – | DQB1*06:02, – | DQA1*01:02,-- |
| DRB1*04, DRB1*13 | DQB1*03 (DQ8), DQB1*06 | DRB1*04:04, DRB1*13:01 | DQB1*03:02, DQB1*06:03 | DQA1*01:03, DQA1*03:01 |
| DRB1*07, DRB1*15 | DQB1*02, DQB1*05 | DRB1*07:01, DRB1*15:01 | DQB1*02:02, DQB1*05:02 | DQA1*01:02, DQA1*02:01 |
| DRB1*07, DRB1*13 | DQB1*02, DQB1*06 | DRB1*07:01, DRB1*13:02 | DQB1*02:02, DQB1*06:04 | DQA1*01:02, DQA1*02:01 |
| DRB1*07, DRB1*-- | DQB1*02, DQB1*03 (DQ9) | DRB1*07:01, — | DQB1*02:02, DQB1*03:03 | DQA1*02:01,-- |
| DRB1*01, DRB1*11 | DQB1*03 (DQ7), DQB1*05 | DRB1*01:01, DRB1*11:01 | DQB1*03:01, DQB1*05:01 | DQA1*01:01, DQA1*05:05 |
| DRB1*12, DRB1*14 | DQB1*03 (DQ7), DQB1*05 | DRB1*12:01, DRB1*14:01 | DQB1*03:01, DQB1*05:03 | DQA1*01:01, DQA1*05:05 |
| DRB1*03, DRB1*11 | DQB1*02, DQB1*05 | DRB1*03:01, DRB1*11:01 | DQB1*02:01, DQB1*05:02 | DQA1*01:02, DQA1*05:01 |
| DRB1*11, DRB1*13 | DQB1*03 (DQ7), DQB1*06 | DRB1*11:04, DRB1*13:01 | DQB1*03:01, DQB1*06:03 | DQA1*01:03, DQA1*05:05 |
| DRB1*09, DRB1*13 | DQB1*02, DQB1*06 | DRB1*09:01, DRB1*13:02 | DQB1*02:02, DQB1*06:04 | DQA1*01:02, DQA1*03:01 |
| DRB1*10, DRB1*15 | DQB1*05, DQB1*06 | DRB1*10:01, DRB1*15:01 | DQB1*05:01, DQB1*06:02 | DQA1*01:01, DQA1*01:02 |
| DRB1*13, DRB1*16 | DQB1*05, DQB1*06 | DRB1*13:01, DRB1*16:01 | DQB1*05:02, DQB1*06:03 | DQA1*01:02, DQA1*01:03 |
| DRB1*01, DRB1*15 | DQB1*05, DQB1*06 | DRB1*01:02, DRB1*15:01 | DQB1*05:01, DQB1*06:02 | DQA1*01:01, DQA1*01:02 |
| DRB1*04, DRB1*11 | DQB1*03 (DQ7), DQB1*03 (DQ8) | DRB1*04:03, DRB1*11:04 | DQB1*03:01, DQB1*03:02 | DQA1*03:01, DQA1*05:05 |
| DRB1*07, DRB1*13 | DQB1*02, DQB1*06 | DRB1*07:01, DRB1*13:02 | DQB1*02:02, DQB1*06:04 | DQA1*01:02, DQA1*02:01 |
| DRB1*11, DRB1*13 | DQB1*03 (DQ7), DQB1*06 | DRB1*11:01, DRB1*13:01 | DQB1*03:01, DQB1*06:03 | DQA1*01:03, DQA1*05:03 |
| DRB1*07, DRB1*11 | DQB1*02, DQB1*03 (DQ7) | DRB1*07:01, DRB1*11:01 | DQB1*02:02, DQB1*03:01 | DQA1*02:01, DQA1*05:03 |
| DRB1*03, DRB1*11 | DQB1*02, DQB1*03 (DQ7) | DRB1*03:01, DRB1*11:01 | DQB1*02:01, DQB1*03:01 | DQA1*05:01, ----------------- |
| DRB1*03, DRB1*15 | DQB1*02, DQB1*06 | DRB1*03:01, DRB1*15:01 | DQB1*02:01, DQB1*06:02 | DQA1*01:02, DQA1*05:01 |
| DRB1*07, DRB1*08 | DQB1*02, DQB1*03 (DQ7) | DRB1*07:01, DRB1*08:03 | DQB1*02:02, DQB1*03:01 | DQA1*02:01, DQA1*06:01 |
| DRB1*01, DRB1*11 | DQB1*03 (DQ7), DQB1*05 | DRB1*01:01, DRB1*11:02 | DQB1*03:01, DQB1*05:01 | DQA1*01:01, DQA1*05:05 |
| DRB1*03, DRB1*15 | DQB1*02, DQB1*06 | DRB1*03:01, DRB1*15:01 | DQB1*02:01, DQB1*06:02 | DQA1*01:02, DQA1*05:01 |
| DRB1*03, DRB1*07 | DQB1*02, DQB1*-- | DRB1*03:01, DRB1*07:01 | DQB1*02:01, DQB1*02:02 | DQA1*02:01, DQA1*05:01 |
| DRB1*01, DRB1*04 | DQB1*03 (DQ7), DQB1*05 | DRB1*01:02, DRB1*04:05 | DQB1*03:01, DQB1*05:01 | DQA1*01:01, DQA1*05:05 |
| DRB1*07, DRB1*13 | DQB1*02, DQB1*03 (DQ7) | DRB1*07:01, DRB1*13:56 | DQB1*02:02, DQB1*03:01 | DQA1*02:01, DQA1*05:05 |
| DRB1*01, DRB1*15 | DQB1*05, DQB1*06 | DRB1*01:01, DRB1*15:01 | DQB1*05:01, DQB1*06:02 | DQA1*01:01, DQA1*01:02 |
| DRB1*04, DRB1*11 | DQB1*03 (DQ7), DQB1*-- | DRB1*04:07, DRB1*11:01 | DQB1*03:01, – | DQA1*03:02, DQA1*05:05 |
| DRB1*01 (DR103), DRB1*13 | DQB1*05, DQB1*06 | DRB1*01:03, DRB1*13:01 | DQB1*05:01, DQB1*06:03 | DQA1*01:01, DQA1*01:03 |
| DRB1*03, DRB1*04 | DQB1*02, DQB1*-- | DRB1*03:01, DRB1*04:05 | DQB1*02:01, DQB1*02:02 | DQA1*03:02, DQA1*05:01 |
| DRB1*04, DRB1*07 | DQB1*02, DQB1*03 (DQ8) | DRB1*04:02, DRB1*07:01 | DQB1*02:02, DQB1*03:02 | DQA1*02:01, DQA1*03:01 |
| DRB1*04, DRB1*08 | DQB1*03 (DQ7), DQB1*03 (DQ8) | DRB1*04:02, DRB1*08:04 | DQB1*03:01, DQB1*03:02 | DQA1*03:01, DQA1*05:05 |
| DRB1*04, DRB1*-- | DQB1*03 (DQ8), DQB1*04 | DRB1*04:02, DRB1*04:06 | DQB1*03:02, DQB1*04:02 | DQA1*03:01, ----------------- |
| DRB1*04, DRB1*-- | DQB1*03 (DQ8), DQB1*-- | DRB1*04:01, DRB1*04:04 | DQB1*03:02, –----------------- | DQA1*03:01, ----------------- |
| DRB1*04, DRB1*07 | DQB1*02, DQB1*03 (DQ8) | DRB1*04:05, DRB1*07:01 | DQB1*02:02, DQB1*03:02 | DQA1*02:01, DQA1*03:01 |
| DRB1*07, DRB1*11 | DQB1*02, DQB1*03 (DQ7) | DRB1*07:01, DRB1*11:04 | DQB1*02:02, DQB1*03:01 | DQA1*02:01, DQA1*05:05 |
| DRB1*07, DRB1*13 | DQB1*02, DQB1*06 | DRB1*07:01, DRB1*13:01 | DQB1*02:02, DQB1*06:03 | DQA1*01:03, DQA1*02:01 |
| DRB1*03, DRB1*13 | DQB1*02, DQB1*03 (DQ7) | DRB1*03:01, DRB1*13:03 | DQB1*02:01, DQB1*03:01 | DQA1*05:01, -- |
| DRB1*11, DRB1*13 | DQB1*03 (DQ7), DQB1*06 | DRB1*11:04, DRB1*13:02 | DQB1*03:01, DQB1*06:04 | DQA1*01:02, DQA1*05:05 |
| DRB1*13, DRB1*16 | DQB1*03 (DQ7), DQB1*-- | DRB1*13:05, DRB1*16:02 | DQB1*03:01, – | DQA1*05:05, -- |
| DRB1*03, DRB1*04 | DQB1*02, DQB1*03 (DQ8) | DRB1*03:01, DRB1*04:05 | DQB1*02:01, DQB1*03:02 | DQA1*03:02, DQA1*05:01 |
| DRB1*15, DRB1*-- | DQB1*06, DQB1*-- | DRB1*15:01, DRB1*15:03 | DQB1*06:02, – | DQA1*01:02,-- |
| DRB1*04, DRB1*13 | DQB1*02, DQB1*06 | DRB1*04:05, DRB1*13:01 | DQB1*02:01, DQB1*06:03 | DQA1*01:03, DQA1*03:01 |
| DRB1*07, DRB1*-- | DQB1*02, DQB1*-- | DRB1*07:01, — | DQB1*02:02, DQB1*02:02 | DQA1*02:01,-- |
| DRB1*01, DRB1*08 | DQB1*04, DQB1*05 | DRB1*01:01, DRB1*08:01 | DQB1*04:02, DQB1*05:01 | DQA1*01:01, DQA1*04:01 |
| DRB1*11, DRB1*15, | DQB1*03 (DQ7), DQB1*06 | DRB1*11:02, DRB1*15:01 | DQB1*03:19, DQB1*06:02 | DQA1*01:02, DQA1*05:05 |
| DRB1*04, DRB1*07 | DQB1*02, DQB1*03 (DQ8) | DRB1*04:05, DRB1*07:01 | DQB1*02:02, DQB1*03:02 | DQA1*02:01, DQA1*03:01 |
| DRB1*01 (DR103), DRB1*07 | DQB1*02, DQB1*05 | DRB1*01:03, DRB1*07:01 | DQB1*02:02, DQB1*05:01 | DQA1*01:01, DQA1*02:01 |
| DRB1*03, DRB1*11 | DQB1*02, DQB1*03 (DQ7) | DRB1*03:01, DRB1*11:01 | DQB1*02:01, DQB1*03:01 | DQA1*05:01, ------------------- |
| DRB1*01, DRB1*15 | DQB1*05, DQB1*06 | DRB1*01:02, DRB1*15:03 | DQB1*05:01, DQB1*06:02 | DQA1*01:01, DQA1*01:02 |
| DRB1*03, DRB1*11 | DQB1*02, DQB1*03 (DQ7) | DRB1*03:01, DRB1*11:01 | DQB1*02:01, DQB1*03:01 | DQA1*05:01, ---------------- |
| DRB1*07, DRB1*09 | DQB1*02, DQB1*03 (DQ9) | DRB1*07:01, DRB1*09:01 | DQB1*02:02, DQB1*03:03 | DQA1*02:01, DQA1*03:01 |
| DRB1*11, DRB1*13 | DQB1*03 (DQ7), DQB1*06 | DRB1*11:04, DRB1*13:01 | DQB1*03:01, DQB1*06:03 | DQA1*01:03, DQA1*05:05 |
| DRB1*07, DRB1*13 | DQB1*02, DQB1*06 | DRB1*07:01, DRB1*13:02 | DQB1*02:02, DQB1*06:04 | DQA1*01:02, DQA1*02:01 |
| DRB1*08, DRB1*11 | DQB1*03 (DQ7), DQB1*04 | DRB1*08:01, DRB1*11:01 | DQB1*03:01, DQB1*04:02 | DQA1*04:01, DQA1*05:05 |
| DRB1*03, DRB1*04 | DQB1*02, DQB1*03 (DQ8) | DRB1*03:01, DRB1*04:04 | DQB1*02:01, DQB1*03:02 | DQA1*03:01, DQA1*05:01 |
| DRB1*07, DRB1*10 | DQB1*02, DQB1*05 | DRB1*07:01, DRB1*10:01 | DQB1*02:02, DQB1*05:01 | DQA1*01:01, DQA1*02:01 |
| DRB1*04, DRB1*-- | DQB1*03 (DQ8), DQB1*-- | DRB1*04:01, DRB1*04:02 | DQB1*03:02, –--------------- | DQA1*03:01, ------------ |
| DRB1*01, DRB1*13 | DQB1*05, DQB1*06 | DRB1*01:02, DRB1*13:01 | DQB1*05:01, DQB1*06:03 | DQA1*01:01, DQA1*01:03 |
| DRB1*03, DRB1*16 | DQB1*02, DQB1*05 | DRB1*03:01, DRB1*16:01 | DQB1*02:01, DQB1*05:02 | DQA1*01:02, DQA1*05:01 |
| DRB1*03, DRB1*13 | DQB1*02, DQB1*06 | DRB1*03:01, DRB1*13:02 | DQB1*02:01, DQB1*06:04 | DQA1*01:02, DQA1*05:01 |
| DRB1*04, DRB1*13 | DQB1*03 (DQ8), DQB1*06 | DRB1*04:04, DRB1*13:02 | DQB1*03:02, DQB1*06:04 | DQA1*01:02, DQA1*03:01 |
| DRB1*04, DRB1*12 | DQB1*03 (DQ7), DQB1*03 (DQ8) | DRB1*04:05, DRB1*12:01 | DQB1*03:01, DQB1*03:02 | DQA1*03:02, DQA1*05:05 |
| DRB1*11, DRB1*13 | DQB1*03 (DQ7), DQB1*-- | DRB1*11:04, DRB1*13:03 | DQB1*03:01, –-------------- | DQA1*05:05, -- |
| DRB1*03, DRB1*04 | DB1Q*02, DB1Q*03 (DQ8) | DRB1*03:01, DRB1*04:01 | DQB1*02:01, DQB1*03:02 | DQA1*03:01, DQA1*05:01 |
| DRB1*03, DRB1*16 | DQB1*02, DQB1*05 | DRB1*03:01, DRB1*16:01 | DQB1*02:01, DQB1*05:02 | DQA1*01:02, DQA1*05:01 |
| DRB1*01, DRB1*16 | DQB1*05, DQB1*-- | DRB1*01:02, DRB1*16:01 | DQB1*05:01, DQB1*05:02 | DQA1*01:01, DQA1*01:02 |
| DRB1*01, DRB1*13 | DQB1*05, DQB1*06 | DRB1*01:01, DRB1*13:01 | DQB1*05:01, DQB1*06:03 | DQA1*01:01, DQA1*01:03 |
| DRB1*15, DRB1*-- | DQB1*06, DQB1*-- | DRB1*15:01, — | DQB1*06:02, – | DQA1*01:02,-- |
| DRB1*04, DRB1*15 | DQB1*03 (DQ8), DQB1*06 | DRB1*04:01, DRB1*15:01 | DQB1*03:02, DQB1*06:02 | DQA1*01:02, DQA1*03:01 |
| DRB1*13, DRB1*16 | DQB1*05, DQB1*06 | DRB1*13:01, DRB1*16:01 | DQB1*05:02, DQB1*06:03 | DQA1*01:02, DQA1*01:03 |
| DRB1*04, DRB1*-- | DQB1*03 (DQ8), DQB1*-- | DRB1*04:01, DRB1*04:02 | DQB1*03:02, – | DQA1*03:01, -- |
| DRB1*03, DRB1*04 | DQB1*02, DQB1*03 (DQ7) | DRB1*03:01, DRB1*04:05 | DQB1*02:01, DQB1*03:01 | DQA1*03:02, DQA1*05:01 |
| DRB1*01, DRB1*07 | DQB1*02, DQB1*05 | DRB1*01:02, DRB1*07:01 | DQB1*02:02, DQB1*05:01 | DQA1*01:01, DQA1*02:01 |
| DRB1*03, DRB1*11 | DQB1*02, DQB1*03 (DQ7) | DRB1*03:01, DRB1*11:01 | DQB1*02:01, DQB1*03:01 | DQA1*05:01, -- |
| DRB1*15, DRB1*-- | DQB1*06, DQB1*-- | DRB1*15:01, — | DQB1*06:02, – | DQA1*01:02,-- |
| DRB1*01, DRB1*15 | DQB1*05, DQB1*06 | DRB1*01:01, DRB1*15:01 | DQB1*05:01, DQB1*06:02 | DQA1*01:01, DQA1*01:02 |
| DRB1*07, DRB1*13 | DQB1*02, DQB1*03 (DQ7) | DRB1*07:01, DRB1*13:03 | DQB1*02:02, DQB1*03:01 | DQA1*02:01, DQA1*05:05 |
| DRB1*03, DRB1*13 | DQB1*02, DQB1*06 | DRB1*03:01, DRB1*13:02 | DQB1*02:01, DQB1*06:09 | DQA1*01:02, DQA1*05:01 |
| DRB1*04, DRB1*15 | DQB1*04, DQB1*06 | DRB1*04:10, DRB1*15:01 | DQB1*04:02, DQB1*06:02 | DQA1*01:02, DQA1*03:01 |
| DRB1*03, DRB1*16 | DQB1*02, DQB1*05 | DRB1*03:02, DRB1*16:02 | DQB1*02:01, DQB1*05:02 | DQA1*01:02, DQA1*05:03 |
| DRB1*15, DRB1*-- | DQB1*06, DQB1*-- | DRB1*15:01, — | DQB1*06:02, DQB1*06:03 | DQA1*01:02,-- |
| DRB1*09, DRB1*11 | DQB1*03 (DQ7), DQB1*03 (DQ9) | DRB1*09:01, DRB1*11:04 | DQB1*03:01, DQB1*03:03 | DQA1*03:02, DQA1*05:05 |
| DRB1*11, DRB1*-- | DQB1*03 (DQ7), DQB1*-- | DRB1*11:01, DRB1*11:03 | DQB1*03:01, – | DQA1*05:03, DQA1*05:05 |
| DRB1*03, DRB1*13 | DQB1*02, DQB1*06 | DRB1*03:01, DRB1*13:02 | DQB1*02:01, DQB1*06:04 | DQA1*01:02, DQA1*05:01 |
| DRB1*03, DRB1*11 | DQB1*02, DQB1*03 (DQ7) | DRB1*03:01, DRB1*11:01 | DQB1*02:01, DQB1*03:01 | DQA1*05:01, -- |
| DRB1*03, DRB1*07 | DQB1*02, DQB1*-- | DRB1*03:01, DRB1*07:01 | DQB1*02:01, DQB1*02:02 | DQA1*02:01, DQA1*05:01 |
| DRB1*03, DRB1*13 | DQB1*02, DQB1*06 | DRB1*03:01, DRB1*13:02 | DQB1*02:01, DQB1*06:09 | DQA1*01:02, DQA1*05:01 |
| DRB1*04, DRB1*07 | DQB1*02, DQB1*03(DQ8) | DRB1*03:02, DRB1*07:01 | DQB1*02:02, DQB1*03:02 | DQA1*02:01, DQA1*03:01 |
| DRB1*01, DRB1*04 | DQB1*03(DQ8), DQB1*05 | DRB1*01:02, DRB1*04:05 | DQB1*03:02, DQB1*05:01 | DQA1*01:01, DQA1*03:01 |
| DRB1*11, DRB1*14 | DQB1*05, DQB1*03(DQ7) | DRB1*11:04, DRB1*14:01 | DQB1*03:01, DQB1*05:03 | DQA1*01:01, DQA1*05:05 |
| DRB1*04, DRB1*08 | DQB1*03 (DQ8), DQB1*04 | DRB1*04:04, DRB1*08:01 | DQB1*03:02, DQB1*04:02 | DQA1*03:01, DQA1*04:01 |
| DRB1*07, DRB1*13 | DQB1*02, DQB1*06 | DRB1*07:01, DRB1*13:01 | DQB1*02:02, DQB1*06:03 | DQA1*01:03, DQA1*02:01 |
| DRB1*07, DRB1*15 | DQB1*02, DQB1*06 | DRB1*07:01, DRB1*15:01 | DQB1*02:02, DQB1*06:03 | DQA1*01:02, DQA1*02:01 |
| DRB1*11, DRB1*16 | DQB1*03(DQ7), DQB1*05 | DRB1*11:01, DRB1*16:01 | DQB1*03:01, DQB1*05:02 | DQA1*01:02, DQA1*05:05 |
| DRB1*07, DRB1*13 | DQB1*06, DQB1*03(DQ9) | DRB1*07:01, DRB1*13:02 | DQB1*03:03, DQB1*06:04 | DQA1*01:02, DQA1*02:01 |
| DRB1*01, DRB1*15 | DQB1*05, DQB1*06 | DRB1*01:02, DRB1*15:01 | DQB1*05:01, DQB1*06:02 | DQA1*01:01, DQA1*01:02 |
| DRB1*07, DRB1*11 | DQB1*02, DQB1*03(DQ7) | DRB1*07:01, DRB1*11:01 | DQB1*02:02, DQB1*03:01 | DQA1*02:01, DQA1*05:05 |
| DRB1*03, DRB1*04 | DQB1*02, DQB1*03(DQ8) | DRB1*03:01, DRB1*04:01 | DQB1*02:01, DQB1*03:02 | DQA1*03:01, DQA1*05:01 |
| DRB1*04, DRB1*13 | DQB1*03(DQ8), DQB1*06 | DRB1*04:05, DRB1*13:02 | DQB1*03:02, DQB1*06:04 | DQA1*01:02, DQA1*03:01 |
| DRB1*01, DRB1*07 | DQB1*02, DQB1*05 | DRB1*01:02, DRB1*07:01 | DQB1*02:02, DQB1*05:01 | DQA1*01:01, DQA1*02:01 |
| DRB1*01, DRB1*11 | DQB1*03(DQ7), DQB1*05 | DRB1*01:02, DRB1*11:01 | DQB1*03:01, DQB1*05:01 | DQA1*01:01, DQA1*05:05 |
| DRB1*03, DRB1*15 |  | DRB1*03:01, DRB1*15:03 | DQB1*02:01, DQB1*06:02 | DQA1*01:02, DQA1*05:01 |
| DRB1*13, DRB1*-- |  | DRB1*13:01, — | DQB1*06:03, – | DQA1*01:02, DQA1*01:03 |
| DRB1*03, DRB1*13 |  | DRB1*03:01, DRB1*13:01 | DQB1*02:01, DQB1*06:03 | DQA1*01:03, DQA1*05:01 |
| DRB1*03, DRB1*13 |  | DRB1*03:01, DRB1*13:01 | DQB1*02:01, DQB1*06:03 | DQA1*01:03, DQA1*05:01 |
| DRB1*13, DRB1*16 | DQB1*05, DQB1*06 | DRB1*13:02, DRB1*16:01 | DQB1*05:02, DQB1*06:04 | DQA1*01:02,-- |
| DRB1*04, DRB1*15 |  | DRB1*04:04, DRB1*15:01 | DQB1*03:02, DQB1*06:02 | DQA1*01:02, DQA1*03:01 |
| DRB1*11, DRB1*14 |  | DRB1*11:01, DRB1*14:01 | DQB1*03:01, DQB1*05:03 | DQA1*01:01, DQA1*05:05 |
| DRB1*03, DRB1*13 | DQB1*02, DQB1*06 | DRB1*03:01, DRB1*13:01 | DQB1*02:01, DQB1*06:03 | DQA1*01:03, DQA1*05:01 |
| DRB1*09, DRB1*15 | DQB1*03 (DQ9), DQB1*06 | DRB1*09:01, DRB1*15:03 | DQB1*03:03, DQB1*06:02 | DQA1*01:02, DQA1*03:01 |
| DRB1*03, DRB1*04 | DQB1*02, DQB1*03 (DQ8) | DRB1*03:01, DRB1*04:05 | DQB1*02:01, DQB1*03:02 | DQA1*03:02, DQA1*05:01 |
| DRB1*01, DRB1*13 | DQB1*05, DQB1*06 | DRB1*01:02, DRB1*13:02 | DQB1*05:01, DQB1*06:09 | DQA1*01:01, DQA1*01:02 |
| DRB1*03, DRB1*11 | DQB1*02, DQB1*03(DQ7) | DRB1*03:01, DRB1*11:04 | DQB1*02:01, DQB1*03:01 | DQA1*05:01, DQA1*05:04 |
| DRB1*07, DRB1*-- | DQB1*02, DQB1*03 (DQ9) | DRB1*07:01, — | DQB1*02:02, DQB1*03:03 | DQA1*02:01,-- |
| DRB1*01, DRB1*03 | DQB1*02, DQB1*05 | DRB1*01:01, DRB1*03:01 | DQB1*02:01, DQB1*05:01 | DQA1*01:01, DQA1*05:01 |
| DRB1*01, DRB1*07 | DQB1*02, DQB1*05 | DRB1*01:02, DRB1*07:01 | DQB1*02:02, DQB1*05:01 | DQA1*01:01, DQA1*02:01 |
| DRB1*01, DRB1*16 | DQB1*05, DQB1*- | DRB1*01:02, DRB1*16:01 | DQB1*05:01, DQB1*05:02 | DQA1*01:01, DQA1*01:02 |
| DRB1*01, DRB1*04 | DQB1*03(DQ8), DQB1*05 | DRB1*01:02, DRB1*04:05 | DQB1*03:02, DQB1*05:01 | DQA1*01:01, DQA1*03:01 |
| DRB1*11, DRB1*14 | DQB1*02, DQB1*05 | DRB1*11:01, DRB1*14:01 | DQB1*02:02, DQB1*05:03 | DQA1*01:01, DQA1*03:01 |
| DRB1*01, DRB1*07 | DQB1*02, DQB1*05 | DRB1*01:01, DRB1*07:01 | DQB1*02:02, DQB1*05:01 | DQA1*01:01, DQA1*02:01 |
| DRB1*13, DRB1*16 | DQB1*05, DQB1*06 | DRB1*13:01, DRB1*16:01 | DQB1*05:02, DQB1*06:03 | DQA1*01:02, DQA1*01:03 |
| DRB1*07, DRB1*-- | DQB1*02, DQB1*03 (DQ9) | DRB1*07:01, — | DQB1*02:02, DQB1*03:03 | DQA1*02:01,-- |
| DRB1*10, DRB1*11 | DQB1*03 (DQ7), DQB1*05 | DRB1*10:01, DRB1*11:01 | DQB1*03:01, DQB1*05:01 | DQA1*01:01, DQA1*05:05 |
| DRB1*04, DRB1*13 | DQB1*03 (DQ7), DQB1*06 | DRB1*04:07, DRB1*13:01 | DQB1*03:01, DQB1*06:03 | DQA1*01:03, DQA1*03:01 |
| DRB1*13, DRB1*-- | DQB1*06, DQB1*-- | DRB1*13:01, DRB1*13:36 | DQB1*06:03, DQB1*06:04 | DQA1*01:02, DQA1*01:03 |
| DRB1*11, DRB1*16 | DQB1*03 (DQ7), DQB1*05 | DRB1*11:01, DRB1*16:01 | DQB1*03:01, DQB1*05:02 | DQA1*01:02, DQA1*05:05 |
| DRB1*07, DRB1*10 | DQB1*02, DQB1*05 | DRB1*07:01, DRB1*10:01 | DQB1*02:02, DQB1*05:01 | DQA1*01:01, DQA1*02:01 |
| DRB1*08, DRB1*14 | DQB1*04, DQB1*05 | DRB1*08:01, DRB1*14:01 | DQB1*04:02, DQB1*05:03 | DQA1*01:01, DQA1*04:01 |
| DRB1*08, DRB1*12 | DQB1*03 (DQ7), DQB1*06 | DRB1*08:03, DRB1*12:01 | DQB1*03:01, DQB1*06:01 | DQA1*01:03, DQA1*05:05 |
| DRB1*03, DRB1*13 | DQB1*02, DQB1*06 | DRB1*03:01, DRB1*13:40 | DQB1*02:01, DQB1*06:03 | DQA1*01:03, DQA1*05:01 |
| DRB1*07, DRB1*15 | DQB1*02, DQB1*06 | DRB1*07:01, DRB1*15:01 | DQB1*02:02, DQB1*06:02 | DQA1*01:02, DQA1*02:01 |
| DRB1*07, DRB1*11 | DQB1*02, DQB1*03 (DQ7) | DRB1*07:01, DRB1*11:01 | DQB1*02:02, DQB1*03:01 | DQA1*02:01, DQA1*05:05 |
| DRB1*11, DRB1*15 | DQB1*03 (DQ7), DQB1*06 | DRB1*11:02, DRB1*15:01 | DQB1*03:19, DQB1*06:02 | DQA1*01:02, DQA1*05:05 |
| DRB1*01, DRB1*13 | DQB1*05, DQB1*06 | DRB1*01:02, DRB1*13:01 | DQB1*05:01, DQB1*06:03 | DQA1*01:01, DQA1*01:03 |
| DRB1*01, DRB1*03 | DQB1*02, DQB1*05 | DRB1*01:02, DRB1*03:01 | DQB1*02:01, DQB1*05:01 | DQA1*01:01, DQA1*05:01 |
| DRB1*01 (DR103), DRB1*13 | DQB1*05, DQB1*06 | DRB1*01:03, DRB1*13:02 | DQB1*05:01, DQB1*06:04 | DQA1*01:01, DQA1*01:02 |
| DRB1*03, DRB1*13 | DQB1*02, DQB1*06 | DRB1*03:01, DRB1*13:01 | DQB1*02:01, DQB1*06:03 | DQA1*01:03, DQA1*05:01 |
| DRB1*08, DRB1*12 | DQB1*03 (DQ7), DQB1*06 | DRB1*08:03, DRB1*12:01 | DQB1*03:01, DQB1*06:01 | DQA1*01:03, DQA1*05:05 |
| DRB1*03, DRB1*07 | DQB1*02, DQB1*02 | DRB1*03:01, DRB1*07:01 | DQB1*02:01, DQB1*02:02 | DQA1*02:01, DQA1*05:01 |
| DRB1*11, DRB1*-- | DQB1*03 (DQ7), DQB1*-- | DRB1*11:02, DRB1*11:03 | DQB1*03:01, DQB1*03:19 | DQA1*05:05, -- |
| DRB1*01 (DR103),DRB1*07 | DQB1*02, DQB1*05 | DRB1*01:03, DRB1*07:01 | DQB1*02:02, DQB1*05:01 | DQA1*01:01, DQA1*02:01 |
| DRB1*07, DRB1*08 | DQB1*02, DQB1*04 | DRB1*07:01, DRB1*08:02 | DQB1*02:02, DQB1*04:02 | DQA1*02:01, DQA1*04:01 |
| DRB1*11, DRB1*15 | DQB1*03 (DQ7), DQB1*06 | DRB1*11:01, DRB1*15:01 | DQB1*03:01, DQB1*06:02 | DQA1*01:02, DQA1*05:05 |
| DRB1*01, DRB1*15 | DQB1*05, DQB1*06 | DRB1*01:01, DRB1*15:01 | DQB1*05:01, DQB1*06:02 | DQA1*01:01, DQA1*01:02 |
| DRB1*01, DRB1*14 | DQB1*03 (DQ7), DQB1*05 | DRB1*01:01, DRB1*14:01 | DQB1*03:01, DQB1*05:01 | DQA1*01:01, DQA1*05:03 |
| DRB1*13, DRB1*-- | DQB1*06, DQB1*-- | DRB1*13:01, — | DQB1*06:03, – | DQA1*01:02, DQA1*01:03 |
| DRB1*04, DRB1*11 | DQB1*03 (DQ7), DQB1*03 (DQ8) | DRB1*04:05, DRB1*11:02 | DQB1*03:02, DQB1*03:19 | DQA1*03:02, DQA1*05:05 |
| DRB1*01, DRB1*03 | DQB1*02, DQB1*05 | DRB1*01:01, DRB1*03:01 | DQB1*02:01, DQB1*05:01 | DQA1*01:01, DQA1*05:01 |
| DRB1*13, DRB1*-- | DQB1*06, DQB1*-- | DRB1*13:01, — | DQB1*06:03, – | DQA1*01:02, DQA1*01:03 |
| DRB1*11, DRB1*13 | DQB1*03 (DQ7), DQB1*-- | DRB1*11:03, DRB1*13:03 | DQB1*03:01, – | DQA1*05:05, -- |
| DRB1*07, DRB1*08 | DQB1*02, DQB1*04 | DRB1*07:01, DRB1*08:01 | DQB1*02:02, DQB1*04:02 | DQA1*02:01, DQA1*04:01 |
| DRB1*07, DRB1*11 | DQB1*02, DQB1*03 (DQ7) | DRB1*07:01, DRB1*11:01 | DQB1*02:02, DQB1*03:01 | DQA1*02:01, DQA1*05:05 |
| DRB1*07, DRB1*09 | DQB1*03 (DQ9), DQB1*-- | DRB1*07:01, DRB1*09:01 | DQB1*03:03, – | DQA1*02:01, DQA1*03:01 |
| DRB1*09, DRB1*16 | DQB1*03 (DQ9), DQB1*05 | DRB1*09:01, DRB1*16:01 | DQB1*03:03, DQB1*05:02 | DQA1*01:02, DQA1*03:01 |
| DRB1*11, DRB1*13 | DQB1*02, DQB1*03(DQ7) | DRB1*11:01, DRB1*13:03 | DQB1*02:02, DQB1*03:01 | DQA1*02:01, DQA1*05:05 |
| DRB1*03, DRB1*07 | DQB1*02, DQB1*- | DRB1*03:01, DRB1*07:01 | DQB1*02:01, DQB1*02:02 | DQA1*02:01, DQA1*05:01 |
| DRB1*01, DRB1*04 | DQB1*03(DQ7), DQB1*05 | DRB1*01:02, DRB1*04:01 | DQB1*03:01, DQB1*05:01 | DQA1*01:01, DQA1*03:01 |
| DRB1*07, DRB1*15 | DQB1*02, DQB1*05 | DRB1*07:01, DRB1*15:01 | DQB1*02:02, DQB1*05:01 | DQA1*01:02, DQA1*02:01 |
| DRB1*07, DRB1*13 | DQB1*02, DQB1*06 | DRB1*07:01, DRB1*13:01 | DQB1*02:02, DQB1*06:03 | DQA1*01:03, DQA1*02:01 |
| DRB1*13, DRB1*-- | DQB1*06, DQB1*-- | DRB1*13:02, — | DQB1*06:04, – | DQA1*01:02,-- |
| DRB1*04, DRB1*08 | DQB1*03 (DQ8), DQB1*04 | DRB1*04:03, DRB1*08:01 | DQB1*03:02, DQB1*04:02 | DQA1*03:01, DQA1*04:01 |
| DRB1*01, DRB1*07 | DQB1*02, DQB1*05 | DRB1*01:01, DRB1*07:01 | DQB1*02:02, DQB1*05:01 | DQA1*01:01, DQA1*02:01 |
| DRB1*03, DRB1*07 | DQB1*02, DQB1*-- | DRB1*03:01, DRB1*07:01 | DQB1*02:01, DQB1*02:02 | DQA1*02:01, DQA1*05:01 |

Supplementary Table 5, Low resolution HLA analysis and estimation of fourth digit in children with T1D (4^th^ digit estimation only in 75 children),

| ***DRB1*** | ***DQB1*** |
| --- | --- |
| DRB1*03:01, DRB1*04 | DQB1*02:01, DQB1*03:02 |
| DRB1*04, - | DQB1*03:02, - |
| DRB1*03, DRB1*04 | DQB1*02:01, DQB1*03:02 |
| DRB1*07, DRB1*13 | DQB1*02, DQB1*06 |
| DRB1*04, DRB1*13 | DQB1*03, DQB1*06 |
| DRB1*03, DRB1*09 | DQB1*02, DQB1*03 |
| DRB1*07, DRB1*08 | DQB1*02, DQB1*04 |
| DRB1*01, DRB1*04 | DQB1*05, DQB1*03 |
| DRB1*01, DRB1*04 | DQB1*05, DQB1*03 |
| DRB1*03, DRB1*04 | DQB1*02, DQB1*03 |
| DRB1*03, DRB1*15 | DQB1*02, DQB1*06 |
| DRB1*04, DRB1*07 | DQB1*02, DQB1*03 |
| DRB1*03, DRB1*07 | DQB1*02, - |
| DRB1*03, DRB1*13 | DQB1*02, DQB1*06 |
| DRB1*03, DRB1*04 | DQB1*02, - |
| DRB1*01, DRB1*03 | DQB1*02, DQB1*05 |
| DRB1*04, DRB1*13 | DQB1*03, - |
| DRB1*03, DRB1*04 | DQB1*02, DQB1*03 |
| DRB1*04, DRB1*07 | DQB1*02, DQB1*04 |
| DRB1*04, DRB1*13 | DQB1*03, DQB1*06 |
| DRB1*04, DRB1*13 | DQB1*03, DQB1*06 |
| DRB1*03, - | DQB1*02, - |
| DRB1*03, - | DQB1*02, - |
| DRB1*03, DRB1*04 | DQB1*02, DQB1*03 |
| DRB1*03, DRB1*04 | DQB1*02, DQB1*03 |
| DRB1*01, DRB1*08 | DQB1*03, DQB1*05 |
| DRB1*03, DRB1*04 | DQB1*02, DQB1*03 |
| DRB1*04, DRB1*13 | DQB1*02, DQB1*06 |
| DRB1*04, - | DQB1*03, - |
| DRB1*01, DRB1*04 | DQB1*03, DQB1*05 |
| DRB1*03, DRB1*04 | DQB1*02, DQB1*03 |
| DRB1*07, DRB1*08 | DQB1*02, DQB1*04 |
| DRB1*03, - | DQB1*02, - |
| DRB1*04, - | DQB1*03, - |
| DRB1*04, DRB1*11 | DQB1*03, - |
| DRB1*03, DRB1*16 | DQB1*02, DQB1*05 |
| DRB1*01, DRB1*03 | DQB1*02, DQB1*05 |
| DRB1*08, DRB1*03 | DRB1*02, DQB1*04 |
| DRB1*03, DRB1*04 | DRB1*02, DQB1*03 |
| DRB1*01, DRB1*04 | DQB1*03, DQB1*05 |
| DRB1*04, DRB1*13 | DQB1*03, DQB1*05 |
| DRB1*04, DRB1*13 | DQB1*03, DQB1*06 |
| DRB1*04, DRB1*08 | DQB1*03, - |
| DRB1*03, DRB1*04 | DQB1*02, DQB1*03 |
| DRB1*03, DRB1*04 | DQB1*02, DQB1*03 |
| DRB1*01, DRB1*04 | DQB1*02 , DQB1*05 |
| DRB1*04, DRB1*16 | DQB1*02, DQB1*05 |
| DRB1*03, DRB1*04 | DQB1*02, DQB1*03 |
| DRB1*03, - | DQB1*02, - |
| DRB1*04, - | DQB1*03, - |
| DRB1*03, DRB1*04 | DQB1*02, DQB1*03 |
| DRB1*01, DRB1*07 | DQB1*02, DQB1*05 |
| DRB1*04, DRB1*07 | DQB1*02, DQB1*03 |
| DRB1*03, DRB1*07 | DQB1*02, - |
| DRB1*03, DRB1*04 | DQB1*02, DQB1*03 |
| DRB1*03, DRB1*04 | DQB1*02, DQB1*03 |
| DRB1*03, DRB1*04 | DQB1*02, DQB1*03 |
| DRB1*03, DRB1*04 | DQB1*02, DQB1*03 |
| DRB1*04, DRB1*07 | DQB1*02, DQB1*03 |
| DRB1*04, DRB1*08 | DQB1*03, DQB1*04 |
| DRB1*01, DRB1*03 | DQB1*05, DQB1*02 |
| DRB1*04, DRB1*07 | DQB1*02, DQB1*03 |
| DRB1*03, DRB1*04 | DQB1*02, - |
| DRB1*03, DRB1*04 | DQB1*02, DQB1*03 |
| DRB1*03, DRB1*04 | DQB1*02, DQB1*03 |
| DRB1*04, DRB1*13 | DQB1*03, - |
| DRB1*03, DRB1*04 | DQB1*02, DQB1*03 |
| DRB1*03, DRB1*15 | DQB1*02, DQB1*06 |
| DRB1*03, DRB1*04 | DQB1*02, - |
| DRB1*03, DRB1*04 | DQB1*02 , DQB1*03 |
| DRB1*03, DRB1*04 | DQB1*02, DQB1*03 |
| DRB1*01, DRB1*03 | DQB1*02, DQB1*05 |
| DRB1*01, DRB1*03 | DQB1*02, DQB1*05 |
| DRB1*01, DRB1*08 | DQB1*04, DQB1*05 |
| DRB1*03, DRB1*04 | DQB1*02, DQB1*03 |
| DRB1*04, DRB1*11 | DQB1*02, - |
| DRB1*07, DRB1*11 | DQB1*02, DQB1*03 |
| DRB1*03, DRB1*16 | DQB1*02, DQB1*05 |
| DRB1*03, DRB1*09 | DQB1*02, - |
| DRB1*15, DRB1*04 | DQB1*05, DQB1*03 |
| DRB1*04, DRB1*07 | DQB1*02, DQB1*03 |
| DRB1*01, DRB1*03 | DQB1*02, DQB1*05 |
| DRB1*03,- | DQB1*02:01, - |
| DRB1*04, - | DQB1*03, - |
| DRB1*01, DRB1*04 | DQB1*03, DQB1*05 |
| DRB1*03, DRB1*04 | DQB1*02, DQB1*03 |
| DRB1*03, DRB1*04 | DQB1*02, DQB1*03 |
| DRB1*03, DRB1*04 | DQB1*02, DQB1*03 |
| DRB1*03, DRB1*04 | DQB1*02, DQB1*03 |
| DRB1*03, DRB1*04 | DQB1*02, DQB1*03 |
| DRB1*04, DRB1*13 | DQB1*03, DQB1*06 |
| DRB1*01, DRB1*04 | DQB1*05:01, DQB1*03:04 |
| DRB1*07, DRB1*09 | DQB1*02, - |
| DRB1*01, DRB1*03 | DQB1*02, DQB1*05 |
| DRB1*04, DRB1*07 | DQB1*02, DQB1*03 |
| DRB1*03, DRB1*04 | DQB1*02, DQB1*03 |
| DRB1*01, DRB1*03 | DQB1*02, DQB1*05 |
| DRB1*03, DRB1*09 | DQB1*02, - |
| DRB1*03, DRB1*04 | DQB1*02, - |
| DRB1*04, DRB1*07 | DQB1*02, DQB1*03 |
| DRB1*04, DRB1*10 | DQB1*05, DQB1*03 |
| DRB1*01, DRB1*04 | DQB1*03, DQB1*05 |
| DRB1*01, DRB1*04 | DQB1*02, DQB1*05 |
| DRB1*01, DRB1*15 | DQB1*05, - |
| DRB1*04, DRB1*07 | DQB1*02, DQB1*03 |
| DRB1*03, DRB1*04 | DQB1*02, DQB1*03 |
| DRB1*01, DRB1*07 | DQB1*02, DQB1*05 |
| DRB1*03, - | DQB1*02, - |
| DRB1*01, DRB1*13 | DQB1*05, DQB1*06 |
| DRB1*04, DRB1*11 | DQB1*03, - |
| DRB1*04, DRB1*15 | DQB1*05, DQB1*03 |
| DRB1*03, - | DQB1*02, - |
| DRB1*04, DRB1*11 | DQB1*02, DQB1*03 |
| DRB1*01, DRB1*04 | DQB1*05, DQB1*03 |
| DRB1*03, DRB1*09 | DQB1*02, - |
| DRB1*04, DRB1*13 | DQB1*03, DQB1*06 |
| DRB1*04, DRB1*07 | DQB1*02, DQB1*03 |
| DRB1*03, DRB1*04 | DQB1*02, DQB1*03 |
| DRB1*04, DRB1*07 | DQB1*02, DQB1*03 |
| DRB1*01, DRB1*04 | DQB1*03, DQB1*05 |
| DRB1*01, DRB1*04 | DQB1*05, DQB1*03:02 (DQ8) |
| DRB1*01:03, DRB1*04 | DQB1*05, DQB1*03:02 (DQ8) |
| DRB1*04, DRB1*07 | DQB1*02, DQB1*03 |
| DRB1*03, DRB1*10 | DQB1*02, DQB1*05 |
| DRB1*04, - | DQB1*03, - |
| DRB1*04, DRB1*11 | DQB1*03:01, DQB1*03:02 |
| DRB1*03, DRB1*08 | DQB1*02, DQB1*04 |
| DRB1*04, DRB1*15 | DQB1*03, DQB1*05 |
| DRB1*03, - | DQB1*02, - |
| DRB1*03, DRB1*07 | DQB1*02, - |
| DRB1*04, - | DQB1*03, - |
| DRB1*03, DRB1*04 | DQB1*02, DQB1*03 |
| DRB1*03, DRB1*04 | DQB1*02, - |
| DRB1*01, DRB1*03 | DQB1*02, DQB1*05 |
| DRB1*03, - | DQB1*02, - |
| DRB1*03, - | DQB1*02, - |
| DRB1*01, DRB1*04 | DQB1*05, - |
| DRB1*03, DRB1*04 | DQB1*02, DQB1*03 |
| DRB1*01, DRB1*07 | DQB1*02, DQB1*05 |
| DRB1*01, DRB1*04 | DQB1*03, DQB1*05 |
| DRB1*01, DRB1*03 | DQB1*02, DQB1*05 |
| DRB1*03, DRB1*04 | DQB1*02, DQB1*03 |
| DRB1*04, - | DQB1*03, - |
| DRB1*03, DRB1*04 | DQB1*02, DQB1*03 |
| DRB1*03, - | DQB1*02, - |
| DRB1*03, DRB1*13 | DQB1*02, DQB1*06 |
| DRB1*03, DRB1*04 | DQB1*02 (DQ2), DQB1*03:02 (DQ8) |
| DRB1*03, DRB1*04 | DQB1*02, DQB1*03:02 (DQ8) |
| DRB1*13, - | DQB1*06, - |
| DRB1*04, DRB1*13 | DQB1*02, DQB1*03:01 |
| DRB1*03, DRB1*04 | DQB1*02 (DQ2), DQB1*03:02 (DQ8) |
| DRB1*01, DRB1*13 | DQB1*05, DQB1*06 |
| DRB1*04, DRB1*07 | DQB1*02, DQB1*03:02 |
| DRB1*03, DRB1*04 | DQB1*02, DQB1*03:02 |
| DRB1*03, DRB1*04 | DQB1*02, DQB1*- |
| DRB1*03, DRB1*- | DQB1*02, DQB1*- |
| DRB1*03, DRB1*13 | DQB1*02, DQB1*06 |
| DRB1*04, DRB1*- | DQB1*03:02, DQB1*- |
| DRB1*03, DRB1*07 | DQB1*02, DQB1*02 |
| DRB1*03, DRB1*- | DQB1*02, DQB1*- |
| DRB1*03, DRB1*04 | DQB1*02, DQB1*03(8) |
| DRB1*03, DRB1*04 | DQB1*02, DQB1*03(8) |
| DRB1*03, DRB1*04 | DQB1*03(8),DQB1*02 |
| DRB1*01, DRB1*08 | DQB1*04, DQB1*05 |
| DRB1*04, DRB1*-- | DQB1*03(8), DQB1*-- |
| DRB1*03, - | DQB1*02, - |
| DRB1*03, DRB1*04 | DQB1*02, DQB1*03:02(8) |
| DRB1*03, DRB1*11 | DQB1*02, DQB1*03:01(7) |
| DRB1*03, DRB1*-- | Ya realizado, DQB1*02, DQB1*02 |
| DRB1*03, DRB1*04 | DQB1*02, DQB1*03:02(8) |
| DRB1*01, DRB1*-- | DQB1*05, DQB1*-- |
| DRB1*04:05, DRB1*13:01 | DQB1*03:02, DQB1*06:03 |
| DRB1*03:01, DRB1*04:05 | DQB1*02:01, DQB1*03:02 |
| DRB1*03:01, DRB1*13:02 | DQB1*02:01, DQB1*06:04 |
| DRB1*04:02, DRB1*04:05 | DQB1*03:02, DQB1*03:02 |
| DRB1*04:05, DRB1*13:02 | DQB1*03:02, DQB1*06:04 |
| DRB1*03:01, DRB1*04:05 | DQB1*02:01, DQB1*03:02 |
| DRB1*03:01, DRB1*04:01 | DQB1*02:01, DQB1*03:02 |
| DRB1*04:04, DRB1*04:05 | DQB1*03:02, DQB1*03:02 |
| DRB1*03:05, DRB1*04:02 | DQB1*02:01, DQB1*03:02 |
| DRB1*04:05, DRB1*11:03 | DQB1*03:01, DQB1*03:02 |
| DRB1*03:01, DRB1*04:05 | DQB1*02:01, DQB1*03:02 |
| DRB1*03:01, DRB1*04:02 | DQB1*02:01, DQB1*03:02 |
| DRB1*0101, DRB1*03:01 | DQB1*02:01, DQB1*05:01 |
| DRB1*01:01, DRB1*03:01 | DQB1*02:01, DQB1*05:01 |
| DRB1*03:01, DRB1*04:05 | DQB1*02:01, DQB1*02:02 |
| DRB1*03:01, DRB1*04:05 | DQB1*02:01, DQB1*03:02 |
| DRB1*01:02, DRB1*03:01 | DQB1*02:01, DQB1*05:01 |
| DRB1*04:05, DRB1*13:01 | DQB1*03:02, DQB1*06:03 |
| DRB1*01:02, DRB1*04:04 | DQB1*03:02, DQB1*05:01 |
| DRB1*03:01, DRB1*04:01 | DQB1*02:01, DQB1*03:02 |
| DRB1*04:05, DRB1*07:01 | DQB1*02:02, DQB1*03:02 |
| DRB1*04, DRB1*13:01 | DQB1*03:01, DQB1*03:03 |
| DRB1*03:01, DRB1*07:01 | DQB1*02:01, DQB1*02:02 |
| DRB1*08:04, DRB1*13:03 | DQB1*03:01, DQB1*03:01 |
| DRB1*03:01, DRB1*04:04 | DQB1*02:01, DQB1*03:02 |
| DRB1*04:01, DRB1*04:05 | DQB1*03:02, DQB1*03:02 |
| DRB1*03:01, DRB1*04:04 | DQB1*02:01, DQB1*03:02 |
| DRB1*03:01, DRB1*04:05 | DQB1*02:01, DQB1*03:02 |
| DRB1*03:01, DRB1*16:01 | DQB1*02:01, DQB1*05:02 |
| DRB1*03:01, DRB1*04:02 | DQB1*'02:01, DQB1*03:02 |
| DRB1*04:02, DRB1*13:01 | DQB1*03:02, DQB1*06:03 |
| DRB1*03:01, DRB1*11:01 | DQB1*02:01, DQB1*03:01 |
| DRB1*03:01, DRB1*04:04 | DQB1*02:01, DQB1*03:02 |
| DRB1*04:05, DRB1*13:01 | DQB1*03:02, DQB1*06:03 |
| DRB1*04:02, DRB1*11:01 | DQB1*03:01, DQB1*03:02 |
| DRB1*01:01, DRB1*03:01 | DQB1*02:01, DQB1*05:01 |
| DRB1*13:02, DRB1*13:02 | DQB1*06:04, DQB1*06:04 |
| DRB1*03:01, DRB1*04:05 | DQB1*02:01, DQB1*03:02 |
| DRB1*07:01, DRB1*13:03 | DQB1*02:02, DQB1*03:01 |
| DRB1*01:02, DRB1*04:05 | DQB1*02:02, DQB1*05:01 |
| DRB1*04:02, DRB1*15:01 | DQB1*03:02, DQB1*05:01 |
| DRB1*04:02, DRB1*04:05 | DQB1*02:02, DQB1*03:02 |
| DRB1*03:01, DRB1*04:05 | DQB1*02:01, DQB1*03:02 |
| DRB1*03:01, DRB1*-- | DQB1*02:01, DQB1*-- |
| DRB1*03:01, DRB1*04:03 | DQB1*02:01, DQB1*03:02 |
| DRB1*04:05, DRB1*13:03 | DQB1*03:01, DQB1*03:02 |
| DRB1*03:01, DRB1*-- | DQB1*02:01, DQB1*-- |
| DRB1*01:02, DRB1*04:05 | DQB1*03:02, DQB1*05:01 |
| DRB1*04:01, DRB1*07:01 | DQB1*02:02, DQB1*03:02 |
| DRB1*01:02, DRB1*04:01 | DQB1*03:02, DQB1*05:01 |
| DRB1*03:01, DRB1*04:01 | DQB1*02:01, DQB1*03:02 |
| DRB1*04:05, DQRB1*10:01 | DQB1*03:02, DQB1*05:01 |
| DRB1*03:01, DRB1*03:01 | DQB1*02:01, DQB1*02:01 |
| DRB1*03:01, DRB1*08:01 | DQB1*02:01, DQB1*04:02 |
| DRB1*03:01, DRB1*04:01 | DQB1*02:01, DQB1*03:02 |
| DRB1*03:01, DRB1*04:01 | DQB1*02:01, DQB1*03:02 |
| DRB1*03:01, DRB1*04:05 | DQB1*02:01, DQB1*03:02 |
| DRB1*03:01, DRB1*13:01 | DQB1*02:01, DQB1*06:03 |
| DRB1*03:01, DRB1*04:04 | DQB1*02:01, DQB1*03:02 |
| DRB1*07:01, DRB1*08:01 | DQB1*02:02, DQB1*04:02 |
| DRB1*07, DRB1*11 | DQB1*02, DQB1*03(7) |
| DRB1*03:01, DRB1*- | DQB1*02, DQB1*- |
| DRB1*03:01, DRB1*04:01 | DQB1*02:01, DQB1*03:02 |
| DRB1*03:01, DRB1*- | DQB1*02:01, DQB1*- |
| DRB1*03:01, DRB1*04:05 | DQB1*02:01, DQB1*03:02 |
| DRB1*03:01, DRB1*04:04 | DQB1*02:01, DQB1*03:02 |
| DRB1*03:01, DRB1*04:05 | DQB1*02:01, DQB1*03:02 |
| DRB1*03:01, DRB1*04:05 | DQB1*02:01, DQB1*03:02 |
| DRB1*04:05, DRB1*07:01 | DQB1*02, DQB1*03(8) |
| DRB1*04:05, DRB1*13:02 | DQB1*03:02, DQB1*06:04 |
| DRB1*03:01, DRB1*04:02 | DQB1*02:01, DQB1*03:02 |
| DRB1*04:01, DRB1*13:01 | DQB1*03:02, DQB1*06:03 |
| DRB1*01:01, DRB1*04:01 | DQB1*03:01, DQB1*05:01 |
| DRB1*03:01, DRB1*15:01 | DQB1*02:01, DQB1*06:02 |
| DRB1*03:01, DRB1^04:04 | DQB1*02:01, DQB1*03:02 |
| DRB1*03:01, DRB1*04:05 | DQB1*02:01, DQB1*03:02 |
| DRB1*03:01, DRB1*- | DQB1*02:01, DQB1*- |
| DRB1*04:05, DRB1*10:01 | DQB1*03:02, DQB1*05:01 |
| DRB1*03, DRB1*13 | DQB1*02, DQB1*06 |
| DRB1*04, DRB1*13 | DQB1*03(8), DQB1*06 |
| DRB1*03, DRB1*04 | DQB1*02, *03(8) |
| DRB1*03, DRB1*04 | DQB1*02, DQB1*03(8) |
| DRB1*03, DRB1*13 | DQB1*02, DQB1*06 |
| DRB1*04, DRB1*13 | DQB1*02, DQB1*03(8) |
| DRB1*01, DRB1*13 | DQB1*05, DQB1*06 |
| DRB1*11, DRB1*- | DQB1*03:01, DQB1*03:19 |
| DRB1*03, DRB1*— | DQB1*02, DQB1*— |
| DRB1*03, DRB1*04 | DQB1*02, DQB1*03(8) |
| DRB1*03, DRB1*04 | DQB1*02, DQB1*03(DQ8) |
| DRB1*03, DRB1*04 | DQB1*02, DQB1*03(DQ8) |
| DRB1*03, DRB1*07 | DQB1*02, DQB1*— |
| DRB1*11, DRB1*16 | DQB1*03(DQ7), DQB1*05 |
| DRB1*03, DRB1*04 | DQB1*02, DQB1*03 (DQ8) |
| DRB1*04, DRB1*09 | DQB1*02, DQB1*03(DQ7) |
| DRB1*01, DRB1*04 | DQB1*03(DQ8), DQB1*05 |
| DRB1*03, DRB1*04 | DQB1*03(DQ7), DQB1*03(DQ8) |
| DRB1*03, DRB1*07 | DQB1*02, DQB1*- |
| DRB1*01, DRB1*13 | DQB1*03:19(DQ7), DQB1*05 |
| DRB1*03, DRB1*- | DQB1*02, DQB1*- |
| DRB1*01, DRB1*03 | DQB1*02, DQB1*05 |
| DRB1*03, DRB1*04 | DQB1*02, DQB1*03(DQ8) |
| DRB1*03, DRB1*04 | DQB1*02, DQB1*03:02 (DQ8) |
| DRB1*01, DRB1*03 | DQB1*02, DQB1*05 |
| DRB1*04, DRB1*08 | DQB1*03:02(DQ8), DQB1*04 |
| DRB1*03, DRB1*- | DQB1*02, DQB1*- |
| DRB1*04, DRB1*11 | DQB1*03(7), DQB1*03(8) |
| DRB1*04, DRB1*13 | DQB1*03(DQ8), DQB1*06 |
| DRB1*04, DRB1*09 | DQB1*02, DQB1*03(DQ8) |
| DRB1*04, DRB1*13 | DQB1*03(DQ8), DQB1*06 |
| DRB1*03, DRB1*04 | DQB1*02, DQB1*03(DQ8) |
| DRB1*03, DRB1*04 | DQB1*02, DQB1*03(DQ8) |
| DRB1*03, DRB1*04 | DQB1*02, DQB1*03(DQ8) |
| DRB1*03, DRB1*- | DQB1*02, DQB1*- |
| DRB1*03, DRB1*11 | DQB1*02, DQB1*03(DQ7) |
| DRB1*04, DRB1*- | DQB1*03(DQ8), DQB1*- |
| DRB1*03, DRB1*07 | DQB1*02, DQB1*- |
| DRB1*04, DRB1*07 | DQB1*03:02(DQ8), DQB1*— |
| DRB1*03, DRB1*04 | DQB1*02, DQB1*03:02 (DQ8) |
| DRB1*04, DRB1*13 | DQB1*03:02 (DQ8), DQB1*06 |
| DRB1*03, DRB1*04 | DQB1*02, DQB1*03:02 (DQ8) |
| DRB1*01, DRB1*07 | DQB1*02, DQB1*05 |
| DRB1*03, DRB1*07 | DQB1*02, DQB1*- |
| DRB1*04, DRB1*13 | DQB1*03:02 (DQ8), DQB1*06 |
| DRB1*04, DRB1*13 | DQB1*03:02 (DQ8), DQB1*06 |
| DRB1*07, DRB1*13 | DQB1*03:02(DQ8), DQB1*06 |
| DRB1*04, DRB1*11 | DQB1*03:01(DQ7), DQB1*03:02(DQ8) |
| DRB1*04, DRB1*— | DQB1*03:02 (DQ8), DQB1*— |
| DRB1*01, DRB1*04 | DQB1*03:02 (DQ8), DQB1*05 |
| DRB1*03, DRB1*- | DQB1*02, DQB1*- |
| DRB1*04, DRB1*07 | DQB1*02, DQB1*03:01 (DQ7) |
| DRB1*03, DRB1*04 | DQB1*02, DQB1*03(DQ8) |
| DRB1*03, DRB1*07 | DQB1*02, DQB1*- |
| DRB1*01, DRB1*04 | DQB1*03(DQ7), DQB1*05 |
| DRB1*07, DRB1*08 | DQB1*02, DQB1*04 |
| DRB1*03, DRB1*— | DQB1*02, DQB1*— |
| DRB1*01 (DR103), DRB1*04 | DQB1*03 (DQ8), DQB1*05 |
| DRB1*01, DRB1*13 | DQB1*05, DQB1*06 |
| DRB1*04, DRB1*13 | DQB1*03(DQ8), DQB1*06 |

Supplementary Table 6, Stratified risk by number of *DRB1* risk alleles

|  | Risk groups | Cases | Controls | OR | 95%CI | Chi-square | P-value |
| --- | --- | --- | --- | --- | --- | --- | --- |
| 2 risk alleles | *03/*04 - *03/*04 | 138 (45%) | 14 (6%) | 12.3 | 6.8-22.2 | 95.4 | <0.00001 |
| 1 risk allele | *03/*04 - *0X | 143 (46%) | 79 (36%) | 1.5 | 1.06-2.1 | 5.3 | 0.02 |
| 0 risk alleles | *0X - *0X | 28 (9%) | 129 (58%) | 0.11 | 0.07-0.18 | 92.9 | 0.00001 |

Supplementary Table 7, Stratified risk by number of *DQB1* risk alleles

|  | Risk groups | Cases | Controls | OR | 95%CI | Chi-square | p-value |
| --- | --- | --- | --- | --- | --- | --- | --- |
| 2 risk alleles | *02/*03 - *02/*03 | 196 | 71 | 3.5 | 2.4-5.1 | 47.5 | <0.00001 |
| 1 risk allele | *02/*03 - *0X | 102 | 109 | 0.48 | 0.3-0.6 | 16.1 | 0.00006 |
| 0 risk alleles | *0X - *0X | 11 | 36 | 0.19 | 0.09-0.37 | 26.6 | <0.00001 |

Supplementary Table 8, HLA risk and protective alleles, haplotypes, or genotypes in different populations,

| Author, year | Participants | Ethnicity | Risk HLA | Protective HLA |
| --- | --- | --- | --- | --- |
| Erlich et al[5], 2008  T1DGC | 607/38 families for a total of 898 T1D patients and 1214 controls, | European/Asian ancestry | DRB1*03:01-DQA1*0501-DQB1*02:01 (OR 3.64)  DRB1*04:05-DQA1*0301-DQB1*03:02 (OR 11.3)  DRB1*04:01-DQA1*0301-DQB*03:02 (OR 8.3)  DRB1*04:02-DQA1*0301-DQB1*03:02 (OR 3.6)  DRB1*04:04-DQA1*0301-DQB1*03:02 (OR 1.5)  DRB1*08:01-DQB1*0401-DQB1*04:02 (OR 1.2) | DRB1*15:01-DQA1*0102-DQB1*06:02 (OR 0.03)  DRB1*14:01-DQA1*0101-DQB1*05:03 (OR 0.02)  DRB1*07:01-DQA1*0201-DQB1*03:03 (OR 0.02)  DRB1*11:04-DQA1*0501-DQB1*03:01 (OR 0.07)  DRB1*13:03-DQA1*0501-DQB1*03:01 (OR 0.08)  DRB1*12:01-DQA1*0501-DQB1*03:01 (OR 0.29) |
| Noble et al[39], 2013  T1DGC | 772 T1D patients and 1641 controls | African Americans | DRB1*03:01-DQA1*05:01-DQB1*02:01(OR 4.74)  DRB1*04:01/02/04/05-DQA1*03:01-DQB1*03:02  (ORs 9.64/ 6.96/ 4.36/ 10.24)  DRB1*07:01-DQA1*03:01-DQB1*02:01(OR 3.42)  DRB1*09:01-DQA1*03:01-DQB1*02:01(OR 3.17)  DRB1*13:03-DQA1*03:01-DQB1*02:01(OR 12.8)  DRB1*16:01-DQA1*01:02-DQB1*05:02(OR 7.47) | DRB1*03:02-DQA1*04:01-DQB1*04:02 (OR 0.16)  DRB1*08:04-DQA1*04:01-DQB1*03:01 (OR 0.22)  DRB1*13:03-DQA1*02:01-DQB1*02:01g (OR 0.2)  DRB1*14:01-DQA1*01:01-DQB1*05:03 (OR 0.04)  DRB1*15:01-DQA1*01:02-DQB1*06:02 (OR 0.06)  DRB1*15:03-DQA1*01:02-DQB1*06:02 (OR 0.11) |
| Ibrahim et al[42], 2021 | 99 T1D patients and 206 healthy controls | Sudanese | DRB1*03:01 (OR 5)  DRB1*04:02 (OR 4.5)  DRB1*04:05 (OR 3.2) | DRB1*10:01 (OR 0.2)  DRB1*15:02 (OR 0.1)  DRB1*15:03 (OR 0.0) |
| Balcha et al[43], 2020 | 236 T1D patients and 200 controls | Amhara (NorthWest Ethiopia) | DRB1*03:01 (OR 2.1)  DRB1*04 (OR 2.4) | DRB1*15 (OR 0.2) |
| Bourhanbour et al[44], 2015 | 51 families, including 90 T1D patients | Moroccan families | DRB1*03:01-DQB1*02:01  DRB1*04:05-DQB1*03:02  (ORs not included in the original article) | DRB1*15-DQB1*06  DRB1* 07, *11, *13 |
| Stayoussef et al[45], 2009 | 88 T1D patients and 112 controls | Tunisia | DRB1*03:01:01-DQB1*02:01 (OR 5.53)  DRB1*04:01:01-DQB1*03:02 (OR 2.89) | DRB1*07:01:01-DQB1*02:01 (OR 0.34)  DRB1*11:01:01-DQB1*03:01:01 (OR 0.24) |
| Fekih et al[46], 2013 | 119 T1D patients and 292 controls | Tunisia | DRB1*03:01-DQB1*02:01  DRB1*04:02-DQB1*03:02  (ORs not included in the original article) | DRB1*07-DQB1*02  DRB1*11-DQB1*03  DRB1*13-DQB1*06  DRB1*15-DQB1*06 |
| Aribi et al[47], 2005 | 91 related subjects: 39 T1D and 52 healthy controls | Algerian | DRB1*4 (OR 2.1)  DRB1*3/4 (OR 1.3) |  |
| Jiang et al[40], 2021 | 361 T1D patients and 500 healthy controls | Chinese | DRB1*03:01-DQA1*05:01-DQB1*02:01(OR 11.7)  DRB1*04:XX-DQA1*03:01-DQB1*03:02 (OR 11.8) (XX =01,04,05)  DRB1*04:05-DQA1*03:03-DQB1*04:01 (OR 2.3)  DRB1*09:01-DQA1*03:02-DQB1*03:03 (OR 1.6) | DRB1*11:01-DQA1*05:05-DQB1*03:01 (OR 0,09)  DRB1*12:02-DQA1*06:01-DQB1*03:01 (OR 0,09) |
| Ikegami et al[41], 2008 |  | Japanese |  | DRB1*15:01-DQB1*06:02 |
| Padma-Malini et al[57], 2018 | 196 T1D patients and 196 healthy controls | Indian (south India) | DRB1*03 (OR 2.8); DQB1*02:01 (OR 2.9); DQB1*02:02 (OR 3.4)  DRB1*04 (OR 3.3); DQB1*03:02 (OR 7.7) | DRB1*10 (OR 0.4); DRB1*15 (OR 0.3)  DQB1*05:02 (OR 0.28); DQB1*06:01 (OR 0.4) |
| Kanga et al [58]`, 2004 | 38 T1D patients and 257 healthy controls | Indian (North India) | DRB1*03 (OR 28.3); DQB1*02:01 (OR 39.3); DRB1*04 (OR 3.6); DRB1*09 (OR 12.7) | DRB1*02 (OR 0.1) |

**References**

57. Harrison J, Priyanka-Tallapragada D, Baptist A, Sharp A, Bhaskar S, Jog K,

et al. Type 1 diabetes genetic risk score is discriminative of diabetes in

non-Europeans: evidence from a study in India. Sci Rep. 2020;10(1):9450.

<https://doi.org/10.1038/s41598-020-65317-1>.

58. Kanga U, Vaidyanathan B, Jaini R, Menon P, Mehra N. HLA haplotypes

associated with type 1 diabetes mellitus in North Indian children. Hum

Immunol. 2004;65(1):47–53.
